# Molecular composition and ultrastructure of Jurassic paravian feathers

**DOI:** 10.1038/srep13520

**Published:** 2015-08-27

**Authors:** Johan Lindgren, Peter Sjövall, Ryan M. Carney, Aude Cincotta, Per Uvdal, Steven W. Hutcheson, Ola Gustafsson, Ulysse Lefèvre, François Escuillié, Jimmy Heimdal, Anders Engdahl, Johan A. Gren, Benjamin P. Kear, Kazumasa Wakamatsu, Johan Yans, Pascal Godefroit

**Affiliations:** 1 Department of Geology, Lund University, 223 62 Lund, Sweden; 2 SP Technical Research Institute of Sweden, Chemistry, Materials and Surfaces, 501 15 Borås, Sweden; 3 Department of Ecology and Evolutionary Biology, Brown University, Providence, Rhode Island 02906, USA; 4 Operational Direction ‘Earth and History of Life’, Royal Belgian Institute of Natural Sciences, 1000 Brussels, Belgium; 5 Department of Geology, University of Namur, 5000 Namur, Belgium; 6 MAX-IV laboratory, Lund University, 221 00 Lund, Sweden; 7 Chemical Physics, Department of Chemistry, Lund University, 221 00 Lund, Sweden; 8 Department of Cell Biology and Molecular Genetics, University of Maryland, College Park, Maryland 20742, USA; 9 Department of Biology, Lund University, 223 62 Lund, Sweden; 10 Department of Geology, Liège University, 4000 Liège, Belgium; 11 Eldonia, 9 Avenue des Portes Occitanes, 3800 Gannat, France; 12 Museum of Evolution, Uppsala University, 752 36 Uppsala, Sweden; 13 Palaeobiology Programme, Department of Earth Sciences, Uppsala University, 752 36 Uppsala, Sweden; 14 Department of Chemistry, Fujita Health University School of Health Sciences, Toyoake, Aichi 470-1192, Japan

## Abstract

Feathers are amongst the most complex epidermal structures known, and they have a well-documented evolutionary trajectory across non-avian dinosaurs and basal birds. Moreover, melanosome-like microbodies preserved in association with fossil plumage have been used to reconstruct original colour, behaviour and physiology. However, these putative ancient melanosomes might alternatively represent microorganismal residues, a conflicting interpretation compounded by a lack of unambiguous chemical data. We therefore used sensitive molecular imaging, supported by multiple independent analytical tests, to demonstrate that the filamentous epidermal appendages in a new specimen of the Jurassic paravian *Anchiornis* comprise remnant eumelanosomes and fibril-like microstructures, preserved as endogenous eumelanin and authigenic calcium phosphate. These results provide novel insights into the early evolution of feathers at the sub-cellular level, and unequivocally determine that melanosomes can be preserved in fossil feathers.

The Middle-Late Jurassic fossil assemblage found in the Tiaojishan Formation of Liaoning Province in northeastern China has yielded unparalleled evidence on the early evolution of birds^1^,^2^,^3^,^4^. Most importantly, the identification of various feather-like integumental appendages in non-avian and stem avialan theropods has illuminated the diversity and distribution of plumage structures during their adaptive transition towards use in flight^4^. Epidermal traces in the Tiaojishan Formation are preserved as either faint impressions or phosphatised and carbonised residues^4^,^5^. The latter were long thought to be a product of keratin-degrading bacteria^6^. However, more recent interpretations have favoured fossilised melanosomes; that is, melanin-bearing cellular organelles responsible in part for the colouration of skin and its structural derivatives^7^. This landmark hypothesis has spawned an entirely new field of exploratory inference into dinosaurian colour^5^,^7^,^8^,^9^,^10^,^11^, behaviour^5^ and physiology^12^.

Nevertheless, it has also met with vivid debate (see ref. 13 for review). This centres on the observation that microbes colonising the epidermal tissues during decay are virtually indistinguishable from the melanosome-like microbodies recognised in fossils^14^,^15^. Such criticism is aggravated by the lack of unequivocal molecular traces from melanic pigments in ancient feathers and feather-like appendages^15^,^16^. Indeed, claims of melanosomes found in the plumage of non-avian dinosaurs and stem avialans have fundamentally relied upon external morphology^5^,^7^,^8^,^9^,^10^,^11^,^12^, but this is demonstrably inadequate for discriminating pigment organelles from pervasive bacteria^15^. Furthermore, chemical data^17^,^18^,^19^,^20^ have proven inconclusive or lacking in specificity^21^, and alleged melanosomes occurring as imprints (‘mouldic melanosomes’^9^) problematically imply that the surrounding substrate was more resistant to degradation than the microbodies themselves^15^. The matrix retaining ‘mouldic melanosomes’ is assumed to be either residual keratin^9^ or ‘remineralized melanin’^5^, yet no attempt has been made to test these hypotheses^15^. An alternative origin might therefore be plausible because melanosome-like impressions are occasionally found in clay minerals, together with silica crystals and other sedimentary grains adjacent to preserved integumentary structures^15^,^20^.

Here we address the unresolved problem of accurately identifying microbodies, imprints and fibrous structures associated with fossilised feather remains via high-resolution imaging and molecular analysis of an exceptionally preserved new specimen (YFGP-T5199, housed in Yizhou Fossil and Geology Park) of the paravian *Anchiornis*^22^. Our results show that multiple local taphonomic pathways incorporating both organic and geochemical agents contributed to the retention of fibrils, eumelanin pigment and eumelanosomes in the integumentary filaments of YFGP-T5199.

## Results

### Fossil specimen and rationale for sample selection

**Figure 1:**
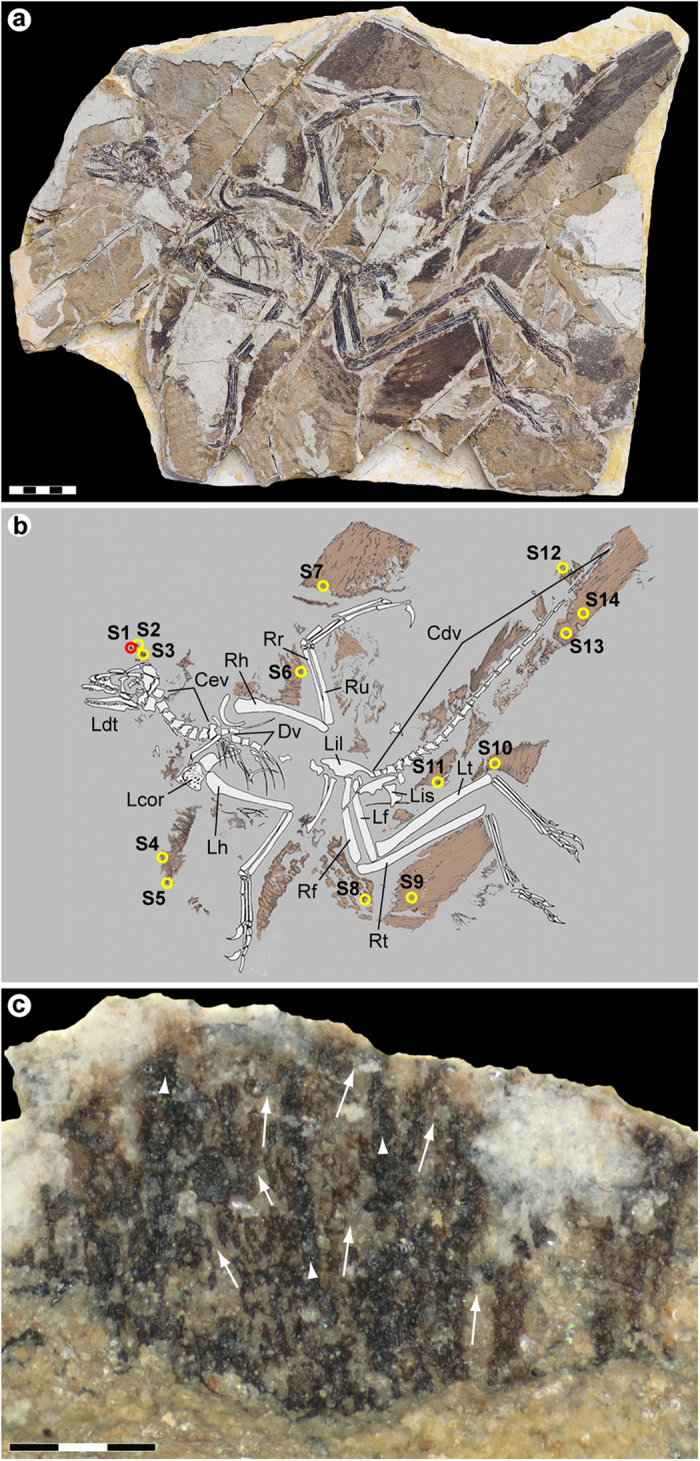
*Anchiornis huxleyi* specimen YFGP-T5199. (**a**) Photographic and (**b**) diagrammatic representation. Numbered circles denote location of plumage samples used for molecular and/or imaging analyses. Red circle (S1) demarcates the ‘forecrown’ sample used as the basis for our investigation; yellow circles (S2–S14) indicate samples used for supportive SEM imaging. Cdv, caudal vertebrae; Cev, cervical vertebrae; Dv, dorsal vertebrae; Lcor, left coracoid; Ldt, left dentary; Lf, left femur; Lh, left humerus; Lil, left ilium; Lis, left ischium; Lt, left tibia; Rf, right femur; Rh, right humerus; Rr, right radius; Rt, right tibia; Ru, right ulna. Scale bar: 5 cm. Photograph by Pascal Godefroit and Ulysse Lefèvre. Drawing by Ulysse Lefèvre. (**c**) Detail of S1 after initial preparation showing darker central strands (arrowheads) with diffuse arrays of filaments branching laterally at acute angles (arrows). Note that the analysed area is still covered by sedimentary matrix (see also Supplementary Fig. S1). Scale bar: 300 μm. Photograph by Johan Lindgren.

An extensively feathered Jurassic paravian referable to *Anchiornis huxleyi* was recovered from the Yaolugou locality in Jianchang County, western Liaoning (see the Supplementary Methods section online). Although initially classified as a non-avian troodontid theropod^1^, recent studies suggest that *Anchiornis* represents a stem avialan, more primitive than *Archaeopteryx*^4^,^23^. The fossil is diagenetically flattened but otherwise essentially complete, comprising an articulated skeleton with plumage remnants forming a dark corona around the bones (Fig. 1a,b). Some integument residues were lost during preparation (see the Supplementary Methods section online); however, patches of feathers and feather-like structures extend along the back half of the skull, lateral to the shoulder girdle, above the pelvic girdle, and along the forelimbs, hind limbs and tail (Fig. 1a,b).

Fourteen samples (S1–S14) ranging in size from about 2 × 2 to 10 × 10 mm were removed from the plumage surfaces (Fig. 1b). One of these (S1) was selected for detailed morphological and molecular examination. S1 was collected some distance above the skull roof (Fig. 1b), in the region of the ‘forecrown’ *sensu* ref. 5 (note that the inferred dorsal crest in *Anchiornis* may be an artefact of preservation^11^,^24^). The sample was considered optimal for investigation because: (1) it showed greyish-brownish colouration indicative of organic remains; (2) was uncovered from a ‘fresh’ sub-surface layer within the sedimentary matrix; (3) produced part and counterpart sub-samples that revealed internal structuring of the filamentous epidermal appendages (Supplementary Fig. S1); and (4) similar ‘crest’ feathers from another *Anchiornis* fossil (see below) have been interpreted as housing pheomelanosomes^5^; that is, spheroid melanosomes dominated by pheomelanin pigment^25^.

### Description of the filamentous epidermal structures and microbodies

The integumentary appendages in S1 superficially resemble feathers of extant birds. They comprise a larger (and darker) central strand (Fig. 1c—arrowheads) with diffuse filamentous arrays branching off laterally at acute angles (Fig. 1c—arrows). In their current, somewhat compressed state, the finer filaments range in width from about 20 to 30 μm, whereas the larger strands measure approximately 40 to 50 μm across.

**Figure 2:**
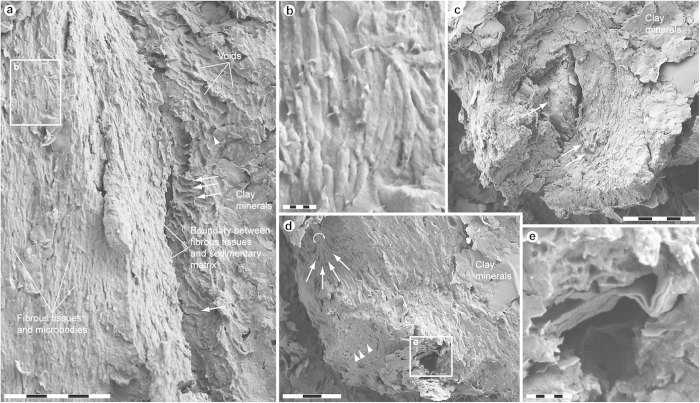
Ultrastructure of YFGP-T5199 ‘forecrown’ feathers. (**a**) FEG-SEM micrograph of fibril-like structures and solid microbodies (left side of image), and densely spaced voids made in the adjacent sedimentary matrix (‘Clay minerals’: right side of image). Note that the fibrous structures and microbodies are roughly aligned in parallel to one another, whereas the imprints are more randomly oriented (those with a longitudinal axis set almost perpendicular to the main direction of the fibrous tissues are marked with arrows). Also note highly variable shape of the voids (one with a sub-circular outline is marked with an arrowhead). Scale bar: 5 μm. (**b**) Enlargement of (**a**) showing aligned, rod-shaped microbodies with rounded termini. Scale bar: 500 nm. (**c**) Oblique transverse view of densely packed fibrils demonstrating their organisation into a larger cylindrical structure, presumably a fibre or barbule. Arrows point at sedimentary infill with randomly oriented elliptical impressions. Scale bar: 5 μm. (**d**) Cross-section of stacked and somewhat ragged fibril-like microstructures with a solid interior (arrowheads). The arrowhead hemi-circle partially encloses a presumed fibril bundle. Note branching patterns (arrows), possibly indicating loss of tension. Scale bar: 3 μm. (**e**) Detail of the area marked in (**d**) showing a wrinkled and partially folded microstructure roughly similar in dimensions to a macrofibril. Scale bar: 500 nm.

Under scanning electron microscopy (SEM) the individual filaments appear as thick, folded sheets of amorphous matter. This morphology is consistent with that of experimentally degraded feathers^26^, and suggests distortion via diagenetic compression and/or elevated temperatures. However, some regions seemingly retain original structure, including continuous layers of densely packed fibrils organised into macrofibrils and/or fibril bundles with a predominantly longitudinal orientation (Fig. 2). Most fibrils are shown in relief, suggesting that they retain at least some of their original three-dimensional form (Fig. 2a); however, extensive folding, wrinkling and branching (Fig. 2d—arrows, e) are congruent with loss of tension during decomposition^27^,^28^. Uneven fracturing has exposed the fibrous material in oblique tangential (Fig. 2a) and transverse views (Fig. 2c,d), revealing that it is part of larger structures (fibres or barbules) with a cylindrical shape (Fig. 2c). The fibrous elements range in size from about 80 nm (fibrils) to >10 μm (fibres/barbules) in diameter. These dimensions are broadly comparable to those recorded for keratinous components of extant bird feathers^27^,^28^,^29^, despite the extent of diagenetic mineralisation (see below).

**Figure 3:**
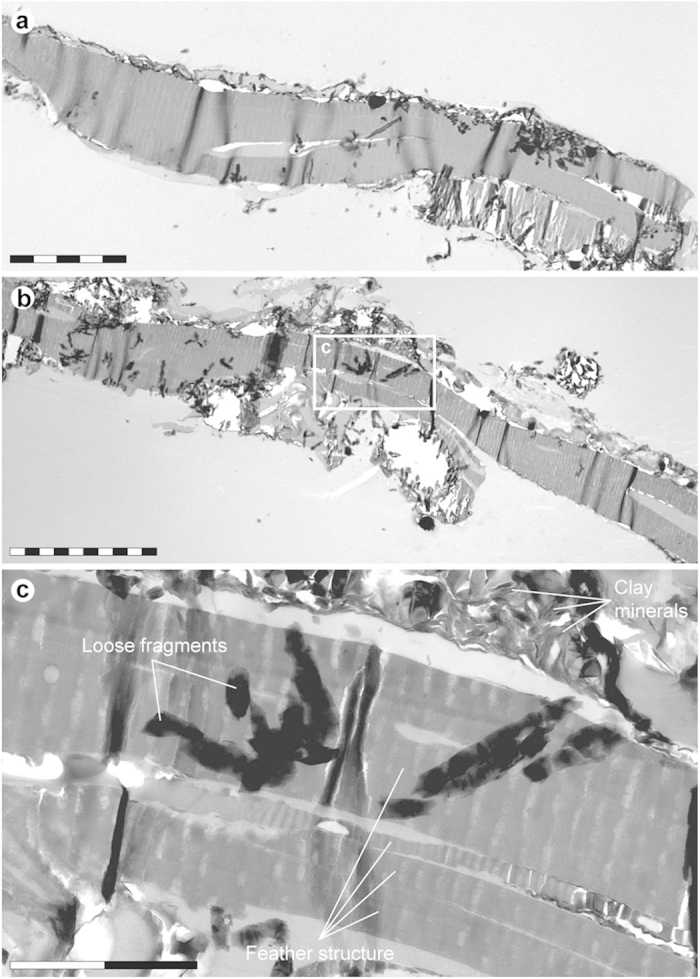
TEM micrographs of YFGP-T5199 ‘forecrown’ feathers. (**a**) Layered microstructures that are superficially similar in both size and organisation to keratin fibrils of extant bird feathers. The corrugated internal texturing and partial rupturing are artefacts of the TEM cutting process. This sample chip was also used for SEM-EDX (Fig. 4) and IR microspectroscopic (Fig. 8) analysis. Scale bar: 5 μm. (**b**) A separate section from the same chip a few micrometres deeper in the fossil feather substrate. Scale bar: 10 μm. (**c**) Enlargement of (**b**) showing details of the feather residues and adhering clay minerals. Scale bar: 2 μm.

Ultrathin sections visualised using transmission electron microscopy (TEM) revealed micrometre-thick, layered structures (Fig. 3), superficially similar to sectioned keratin fibrils of extant feathers (see ref. 15, Fig. 1a).

Stacks of elongate microbodies are locally seen tightly adhering to, partially embedded in, or even merged with the fibrous substrate (Fig. 2a,b). These are crudely aligned in parallel to one another, and their overall orientation follows that of the fibrous tissues (Fig. 2a,b). Individual elements are rod-shaped with rounded termini, and substantially longer than wide. Most of these rods are straight or gently bent (Fig. 2b); however, others are strongly flexed (Supplementary Fig. S2d,e—arrow).

Within the matrix immediately adjacent to the fibrous tissues are densely spaced imprints infesting either a eumelanin/calcium phosphate residue (see discussion below and Supplementary Fig. S2c,d,f), clay minerals (Fig. 2a) or microcrystalline aggregates (Fig. 2c and Supplementary Fig. S2h). Some impressions correspond in shape and size to the rod-shaped microbodies (Supplementary Fig. S2d,f,g). Additionally, they show similar alignment (Supplementary Fig. S2d,f,g), and rod-like elements are even retained in a few imprints (Supplementary Fig. S2d,e—arrow). Other impressions are morphologically more diverse, ranging from ovoid to elongate, and they are also more randomly oriented (sometimes with their long axis set almost perpendicular to that of the fibrous tissues; Fig. 2a—arrows and Supplementary Fig. S2h).

Ultimately, while the morphological and organisational similarities of the fibrous structures and microbodies to those of degraded feather keratin and remnant eumelanosomes are striking (Supplementary Fig. S3; see also refs 27,28,30,31), integration of chemical data is necessary to discriminate between endogenous residues and exogenous microorganisms that may occur associated with decaying keratinaceous substrates (see ref. 30, fig. 6).

### Elemental and molecular analyses

**Figure 4:**
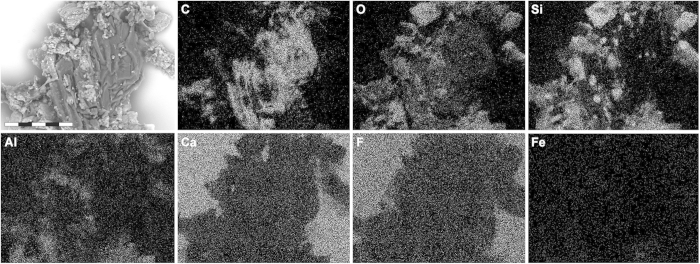
Single-element SEM-EDX maps of YFGP-T5199 ‘forecrown’ feathers. White indicates high intensity whereas black indicates low intensity. Note relatively high levels of carbon (**C**) in the fossil feather material, whereas the sediment is dominated by silica (**Si**) and oxygen (**O**), with minor quantities of aluminium (**Al**) and only trace amounts of iron (**Fe**). Intensities from calcium (**Ca**) and fluoride (**F**) derive from the underlying spectrophotometric window (for IR microspectroscopic analysis). Scale bar: 50 μm.

**Figure 5:**
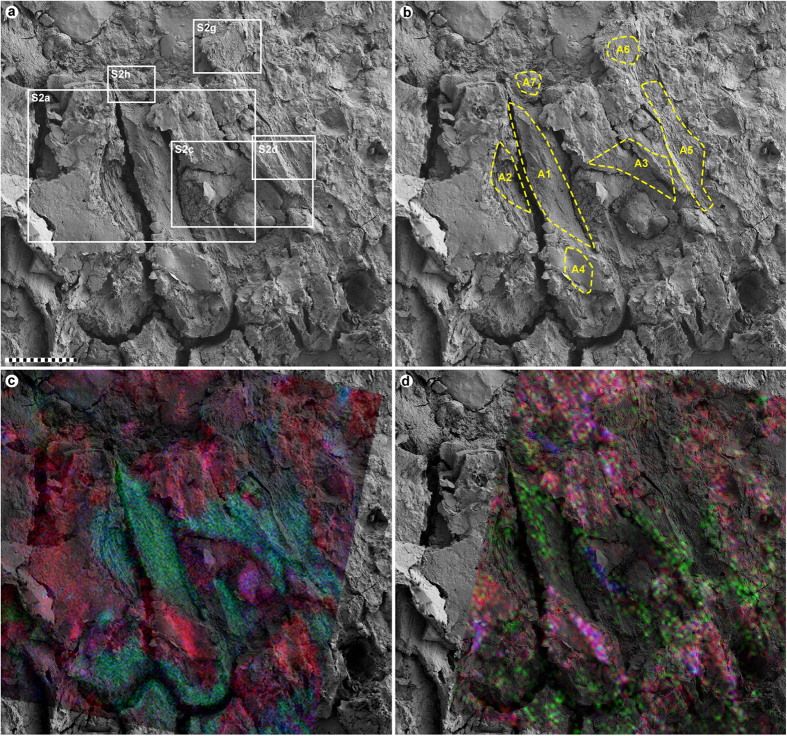
FEG-SEM and ToF-SIMS micrographs of YFGP-T5199 ‘forecrown’ feathers. (**a**) FEG-SEM micrograph of feather material and surrounding sediments. Close-up images of the delimited areas are shown in Supplementary Fig. S2. Scale bar: 20 μm. (**b**) Same image as in (**a**). Stippled yellow lines mark areas (A1–A7) from which the spectra in Figs 6 and 7 and Supplementary Figs S5 and S7 were collected. (**c**) A semi-transparent negative ion image showing the spatial distribution of peaks characteristic of eumelanin (green), phosphate (blue) and silica (red) superimposed onto the FEG-SEM image (see also Supplementary Fig. S4). (**d**) A semi-transparent positive ion image showing the spatial distribution of peaks corresponding to calcium phosphate (green), potassium (blue) and aluminium + silicon (red) superimposed onto the FEG-SEM image (see also Supplementary Fig. S6).

**Figure 6:**
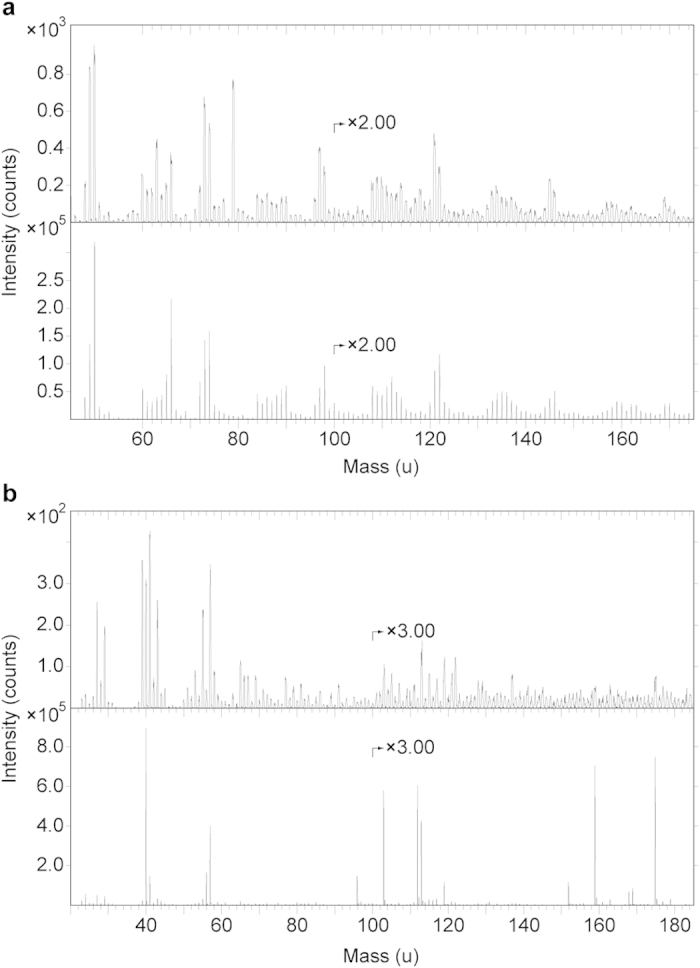
ToF-SIMS spectra from area A1 together with eumelanin and hydroxyapatite. (**a**) Negative ion ToF-SIMS spectra from A1 (top) and synthetic eumelanin (bottom). Note that all major peaks in the synthetic eumelanin spectrum are also present in the fossil spectrum, and that they occur with approximately the same relative signal intensity distribution (see also Supplementary Table S1), indicating the presence of significant amounts of eumelanin at the surface of the fibrous tissues. Additional peaks in the fossil spectrum originate from phosphate (PO_2_^−^ and PO_3_^−^ at 63 and 79 u, respectively) and silicate-related ions (SiO_2_^−^, SiO_3_^−^ and SiO_3_H^−^ at 60, 76 and 77 u, respectively). (**b**) Positive ion ToF-SIMS spectra from A1 (top) and hydroxyapatite (bottom). Note characteristic calcium phosphate peaks at 103, 159 and 175 u in the fossil spectrum, corresponding to CaPO_2_^+^, Ca_2_PO_3_^+^ and Ca_2_PO_4_^+^, respectively (see Supplementary Table S2 for peak assignments).

Energy-dispersive X-ray microanalysis (EDX) identified carbon as the primary component in the integumentary remains, which suggests an organic source (Fig. 4). In addition, time-of-flight secondary ion mass spectrometric (ToF-SIMS) imaging analysis detected negative ions characteristic of melanic pigments localised specifically to areas with fibrous tissues and embedded microbodies (e.g., A1–A3; Figs 5b,c and 6a and Supplementary Figs S2a–e, S4a,e, S5). Detailed comparisons with modern reference samples further revealed that all ‘characteristic’ peaks of the eumelanin molecular structure were present in the fossil spectra, with considerable agreement in both mass position and relative signal intensity distribution (Fig. 6a and Supplementary Table S1). In contrast, the surrounding sediment yielded mainly silica-related negative ions indicative of silicate-rich minerals (e.g., A4; Fig. 5b,c and Supplementary Figs S2a, S4b,e, S5). Phosphate-containing ions were also encountered over the entire surface, but at significantly higher intensities in the melanin-dominated areas (Fig. 5c and Supplementary Fig. S4d).

Positive ion mode spectra generated directly from the feather residues showed increased intensity of calcium phosphate-related ions, indicating preferential localisation of this mineral to the fibrous tissues and microbodies (e.g., A1–A3; Figs 5b,d and 6b and Supplementary Figs S6a,e, S7). Mineral-related ions, including aluminium, silicon, magnesium, and potassium, were found in the adjacent matrix (e.g., A4; Fig. 5b,d and Supplementary Figs S6b–e, S7); these probably denote illite group clay minerals^32^, as intimated by the TEM imaging (Fig. 3c).

ToF-SIMS spectra from areas with microbody imprints (e.g., A5–A7; Fig. 5 and Supplementary Figs S2c,d,f–h, S4–S7) detected varied molecular compositions, ranging from melanin/calcium phosphate-dominated residues (A5) to silicate minerals (A6 and A7), incorporating regionalised intensities of calcium phosphate-related ions (A6).

**Figure 7:**
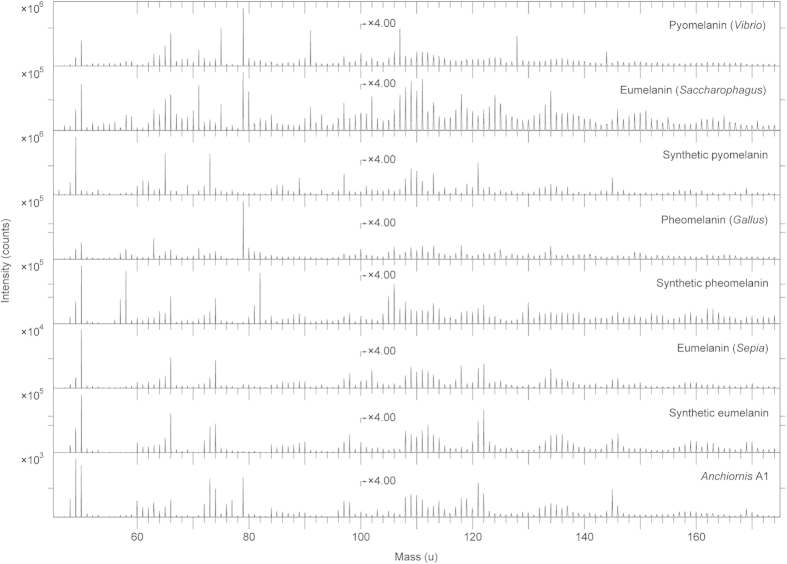
ToF-SIMS spectra acquired from melanin reference samples and A1. Negative ion ToF-SIMS spectra from various melanin standards and reference samples together with the spectrum from area A1. All spectra were acquired with the ToF-SIMS instrument optimised for high mass resolution. The prominent peak at 79 u (PO_3_^−^) in the spectra from pyomelanin (*Vibrio*), eumelanin (*Saccharophagus*) and pheomelanin (*Gallus*) derives from phosphate-containing contaminants, most likely originating from the melanin extraction and/or purification process.

Comparative analyses were undertaken on synthetic and natural variants of eumelanin and pheomelanin, keratin, two peptidoglycans, five hopanoids, three porphyrins, and three microbial mats^33^,^34^,^35^. We also examined a chemically derived pyomelanin, as well as pyomelanin from the bacterium *Vibrio cholerae* and eumelanin from the bacterium *Saccharophagus degradans* (Fig. 7). Based on these data, the fossil melanin from YFGP-T5199 displayed closest agreement with animal eumelanin (Fig. 7). Spectra acquired from the microbial melanins were also compatible in their ‘characteristic’ eumelanin peaks at 73, 74, 97, 98, 121, 122, 145, and 146 u; importantly though, these were significantly different in their relative abundances. The synthetic pyomelanin spectrum lacked all peaks corresponding to nitrogen-containing ions, including those indicative of eumelanin at 50, 66, 74, 98, 122, and 146 u.

Minor contributions from sulfur-containing organics were identified in the spectra from YFGP-T5199, including C_n_NS^−^ ions at 58 (n = 1), 82 (n = 3) and 106 (n = 5) u, and C_n_HS^−^ ions at 57 (n = 2), 81 (n = 4) and 105 (n = 6) u. These peaks were likewise prominent in the synthetic and natural pheomelanin samples (Fig. 7), and showed significant co-localisation with ‘typical’ eumelanin-related peaks (Supplementary Fig. S4a,c). However, the sulfur-containing, possible pheomelanin-related peaks were conspicuously weak in the fossil spectra (Figs 6a and 7), thus preventing confident determination of pheomelanin or diagenetic incorporation of sulfur into the eumelanin molecular structure (as has been previously suggested for other fossil eumelanins^33^,^35^).

No bacterial peptidoglycans or hopanoids were detected, and proteinaceous components consistent with keratins were also absent. Lastly, there were no signs of consolidants and/or preservatives that might potentially compromise the chemical integrity of the sample.

**Figure 8:**
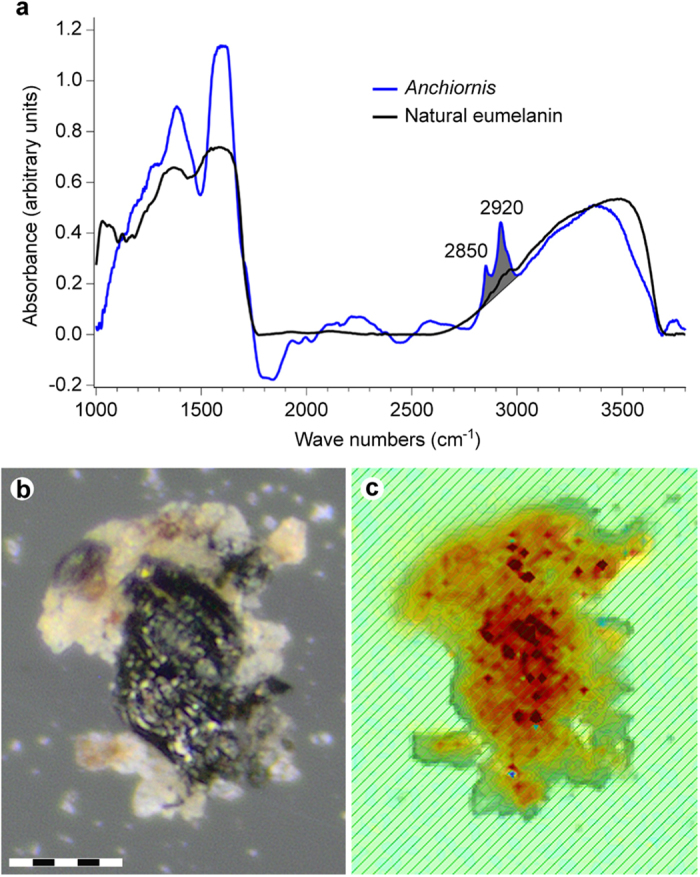
IR absorbance data from YFGP-T5199 ‘forecrown’ feathers. (**a**) Single point IR spectrum recorded from the lower part of the sample chip illustrated in (**b**,**c**). Broad-band absorbance occurs in the 900–1800 and 2500–3700 cm^−1^ regions, consistent with natural eumelanin. The YFGP-T5199 spectrum was recorded using transmission mode and a 15 × 15 μm aperture to reduce sediment contributions. The C-H stretch region used for the absorbance imaging in (**c)** is shaded in grey. (**b**) Optical and (**c**) IR absorbance image (superimposed onto an optical image), the latter recorded by a focal plane array detector (see the Supplementary Methods section online). The IR image is based on the absorbance of the C-H stretches in the 2820–3000 cm^−1^ region from 2,760 individual spectra (the absorbance increases from yellow to red). The C-H stretches are associated with eumelanin-like spectra, which in conjunction with the lack of other organics on the sample chip (as evidenced by ToF-SIMS), suggest derivation primarily from eumelanin residues. Note that the spatial distribution of the C-H stretch absorbance intimately follows the blackish feather material (see consistency with the carbon distribution recorded by SEM-EDX; Fig. 4). Scale bar: 50 μm.

These results were corroborated by IR microspectroscopic measurements, which produced localised absorbance consistent with natural eumelanin, albeit with minor contributions from the surrounding sedimentary matrix (Fig. 8).

## Discussion

The microscopic organisation of the epidermal remains in YFGP-T5199 closely resembles decayed keratin fibrils and eumelanosomes found in extant bird feathers, especially after selective biodegradation of the amorphous polymer matrix^27^,^28^,^30^,^31^. The exceptional morphological fidelity of these filamentous appendages also reveals a fibrillar hierarchy reminiscent of the rachis and barb cortex^27^, which may have imparted a flexural stiffness to the ‘forecrown’ feathers in YFGP-T5199. The mechanical architecture of paravian feathers can thus be shown to extend beyond gross macroanatomy^36^,^37^, to a sub-cellular level of biological organisation.

Multiple independent lines of evidence advocate a eumelanosome origin for the rod-shaped microbodies in the epidermal tissues: (1) the presence of animal eumelanin; (2) the size, shape, distribution, and parallel alignment, which unlike bacterial cells do not form serial chains indicative of microbe fission (see ref. 15, figs 1e and 2a, S1c2 and ref. 30, fig. 6); and (3) the embedment within fibril-like structures similar to feather keratin. Yet despite this striking morphological resemblance, we failed to detect any proteinaceous components indicative of keratins. Instead, the fibrous tissues consisted of eumelanin and calcium phosphate, the latter possibly derived from mineral replacement. Indeed, post-burial melanin leakage might have provided a mechanism for either epidermal tissue stabilisation or replication in YFGP-T5199 that was further facilitated by the rapid growth of authigenic minerals^38^,^39^,^40^. Early mineralisation is a common way of preserving labile soft tissues^38^,^39^,^40^, and often involves calcium phosphate in fossilised melanic and/or keratinous structures, including ink sacs^41^,^42^, feathers^5^,^43^ and claw sheath material^44^.

The association of biomolecules with a mineral substrate is thought to increase the preservation potential of organic compounds, either via adsorptive inhibition of autolysis in decay-inducing enzymes, or by fixation of mineral ions into stabile organometallic complexes that impede molecular breakdown^44^,^45^. Polymer-calcium phosphate^39^,^40^,^44^ and/or polymer-clay^38^,^45^,^46^,^47^ interactions could thus be responsible for the retention of eumelanin molecules in YFGP-T5199. Regardless, eumelanin itself is resistant to decay^48^ because of its extremely dense and insoluble polymer composition that is both antimicrobial and chemically robust^49^,^50^, even in comparison with keratin^9^. Furthermore, mature melanosomes are essentially solid aggregations of melanin, which is polymerised onto an insoluble amyloid fibril scaffolding^51^,^52^, thus imparting an architectural stability that likely allows these specialised organelles to persist in the fossil record.

Some imprints observed in the matrix bordering the fibrous microstructures (e.g., A5) are reasonably interpreted as external moulds of pigment organelles based on their dimensional, distributional and chemical compatibility with the rod-like microbodies interpreted here as remnant eumelanosomes (Fig. 5 and Supplementary Fig. S2d–f), and might have been derived via sample preparation or disruption – e.g., impression from microbodies located on the counterpart (a scenario experimentally shown to produce melanosome imprints in the keratin matrix of modern feathers^8^).

A melanosome origin is also plausibly inferred for other mouldic structures in YFGP-T5199, including those exposed in A6 (note the continuous alignment of the impressions in A5 and A6; Fig. 5b). However, the molecular composition in this region broadly corresponds to that of the host rock (albeit with an increased amount of calcium phosphate), indicating mould formation primarily by aluminosilicate clays. It is therefore possible that local, rapid nucleation and precipitation of clay minerals proceeded in synchrony with the decomposition of the keratinous feather material, thereby encapsulating more stabile organic structures (such as the eumelanosomes) within clay nanofabrics. At a later stage, the entombed organelles also decayed, leaving hollow void spaces that for unknown reasons remained empty (assuming that these melanosome ‘pseudomorphs’ are not negatives from positive reliefs on the counterpart slab).

The affinity of other imprints is more enigmatic, including those in A7 (Supplementary Fig. S2h). Not only are these voids morphologically more diverse, but they are also highly disorganised in comparison to the solid eumelanosomes (Fig. 2a and Supplementary Fig. S2h). Spatially, these impressions are also seemingly restricted to aluminosilicate clays and microcrystalline clay aggregates along the bedding plane (Fig. 2a and Supplementary Fig. S2h). We were unable to locate any three-dimensional microstructures matching the imprints, implying that maker(s) of these moulds were less resistant to decay than the eumelanosomes.

In modern feathers, melanosomes can be organised into discrete layers where individual organelles are either consistently aligned or more erratically oriented with only local uniformity in directions^8^,^53^. Thus, the fossil imprints may comprise moulds of melanosomes that exhibit more variability in shape, and originated from the outer cortex (which can exhibit less melanosome alignment^8^). These organelles may also be more prone to degradation because of greater exposure. Alternatively, preservational biases and diagenesis could potentially modify the appearance of fossil structures^46^,^54^, although this would require the pigment organelles to be transformed not only in size (as has been previously demonstrated^26^), but also in shape and orientation during the fossilisation process (assuming that they were originally aligned in approximate parallel with the bedding plane).

On the other hand, colonies of keratin-degrading microorganisms often comprise a consortium of taxa, resulting in a mixture of shapes and sizes^30^. They are also inherently associated with decaying feathers, and can form clustering patterns where neighbouring cells are oriented in common directions (see ref. 30, Fig. 6). Furthermore, because some decay is necessary to initiate mineralisation^39^, and because calcium phosphate precipitation can be microbially induced^40^,^55^, a microorganismal mediator could be rationally invoked in the partial replacement of the feather material. This, coupled with morphological evidence of fossilised microbes^56^, has implications for the interpretation of the more enigmatic imprints observed in the matrix bordering the fibrous microstructures in YFGP-T5199 (Fig. 2a and Supplementary Fig. S2h). Given the extensive fossil record of microorganisms^56^,^57^, and that some of these voids were not associated with an animal eumelanin molecular signature (e.g., A7), a microbial origin for these particular imprints cannot be excluded. Accordingly, we argue caution against interpreting all fossilised microbodies and impressions as melanosomes, and reconstructing plumage colours based on morphology alone.

An interesting aspect of the fossilised melanosomes in YFGP-T5199 is their exclusively elongate shape (Fig. 2b and Supplementary Fig. S2e). This deviates markedly from the relatively stocky microbodies reported from the ‘forecrown’ feathers of another referred specimen of *A*. *huxleyi* (BMNHC PH828, housed in Beijing Museum of Natural History)^5^. With a few possible exceptions (Fig. 2a—arrowhead), we also detected no pheomelanosome-like structures (see ref. 5). Several explanations might account for these discrepancies, including ontogeny, intraspecific variability and sexual dimorphism, as well as taphonomy, and/or sampling (that is, different regions within a multi-coloured crest). We also have to entertain the possibility that: (1) YFGP-T5199 and BMNHC PH828 represent different taxa; (2) integumentary melanosomes intermingle with melanosomes from other parts of the body in one of the two specimens; and (3) the microbodies and impressions reported by Li *et al.*^5^ and us are different structures altogether.

Indeed, BMNHC PH828 is considerably smaller (~60% by ulna and tibiotarsus length) than YFGP-T5199, and hence it may represent an earlier ontogenetic stage of *A*. *huxleyi*. Furthermore, YFGP-T5199 possesses a uniquely short dorsodistal process of the ischium (see the Supplementary Methods section online), which suggests the possibility of intraspecific and/or sexual differences that may also be reflected in the expressed colour pattern. However, the referral of BMNHC PH828 to *A*. *huxleyi* is problematic, and we cannot exclude that this specimen represents a closely related but different paravian taxon (see the Supplementary Methods section online).

Additionally, while it has been shown that increased temperature and pressure can reduce the size of melanosomes^26^, such alterations may not also include shape (but also see ref. 10). Taphonomy, therefore, presumably cannot account for the different microstructures seen in YFGP-T5199 and BMNHC PH828. Even though melanosomes can potentially disperse during decay^58^, the microbodies in S1 are located deeply within a fibrous matrix interpreted as fossilised keratin. We thus conclude that the melanosomes most feasibly derive from the ‘forecrown’ feathers of YFGP-T5199 as opposed to other dermal tissues and/or internal organs (but whether this is also true for BMNHC PH828 has yet to be determined).

Although a re-investigation of the affinity and preservation of BMNHC PH828 is beyond the scope of this study, we note that the ‘pheomelanosome’ imprints reported by Li *et al.* (ref. 5, Fig. S5) are preserved in sedimentary grains rather than recognisable feather traces, similar to our more enigmatic impressions. Thus, these imprints may also represent either remobilised melanosomes or non-melanosome microstructures.

Our integrated structural and direct chemical approach provides compelling evidence that eumelanosomes and endogenous eumelanin pigment are preserved in the feather remains of YFGP-T5199. This result adds to a growing chronicle of molecular eumelanin detection in fossils^33^,^35^,^42^,^59^,^60^, and demonstrates the aptitude of rigorous experimental techniques for identifying ancient biomolecules and their use in characterising ‘palaeo-colours’.

## Methods

Fourteen feather samples (denoted S1–S14) were removed from YFGP-T5199 using either a sterile scalpel or a hand-operated saw. One sample (S1) selected for molecular analysis was triple-washed successively in acetone, 96% ethanol and Milli-Q water to remove potential contaminants from human handling. The sample was then dried under a hood, wrapped loosely in fresh aluminium foil and stored in isolation inside a sealed sterile glass container. ‘Fresh’ feather material was exposed prior to analysis by removing encasing sediment with a sterile scalpel; the resulting sample chip was subsequently split into part and counterpart pieces. Fresh aluminium foil was used to cover all work areas, and sterile surgical gloves were used during all handling and preparation. Our treatment procedure was identical for all modern reference samples. All experiments were repeated in order to validate the results.

### SEM and FEG-SEM

Initial screening of S1 was performed using a Hitachi S-3400N SEM on the uncoated sample under low vacuum, and the elemental composition was determined via elemental mapping using EDX analysis (1900 sec scanning time at 15 keV, 62.0 μA and a working distance of 10 mm). Following ToF-SIMS analysis, S1 was sputter-coated with a gold/palladium mixture and re-examined using a Zeiss Supra 40VP FEG-SEM (2 keV, working distance 3–5 mm, Everhart-Thornley secondary electron detector). Samples S2–S14 were sputter-coated with gold or gold/palladium and analysed using an environmental QUANTA 200 (FEI) SEM and a Zeiss Supra 40VP FEG-SEM.

### TEM

Fossil feather material was removed from S1 using a sterile scalpel and placed in pure alcohol. The alcohol was then replaced with acetone, and stepwise substituted with epoxy resin (AGAR 100 Resin kit, R1031) to fully infiltrate the remnant tissues. The epoxy was left to polymerise at 60 °C for 48 h. Infiltrated sub-samples were trimmed with a razor blade and then 1.5 μm thick sections were cut using a glass knife mounted on an ultrotome (Leica Ultracut UCT). A diamond knife was employed for the ultra-thin sectioning at 50 nm, after which slices were fixed to pioloform-coated copper grids. These were inserted into a JEOL JEM-1230 transmission electron microscope run at 80 kV. Areas of interest were photographed using a Gatan MultiScan 794 CCD camera.

### ToF-SIMS

The ‘freshly’ prepared part and counterpart sub-samples of S1 were fixed on a metal block using double-sided tape (Supplementary Fig. S1) and then immediately inserted into a ToF-SIMS IV instrument (IONTOF GmbH). ToF-SIMS analyses in the static SIMS mode were performed using 25 keV Bi_3_^+^ primary ions and low energy electron flooding for charge compensation. High mass resolution data were acquired in the bunched mode (m/∆m ~ 5000) at a spatial resolution of ~3–4 μm, whereas high image resolution data were obtained without bunching (m/∆m ~ 300, spatial resolution ~0.2–0.5 μm); in both cases at 256 × 256 pixels. The coordinates for all positions investigated were monitored in order to allow for subsequent FEG-SEM analysis of the same areas.

### IR microspectroscopy

Fossil tissues and sediments were removed from S1 using a sterile scalpel, suspended in Milli-Q water, and then placed on sterile CaF_2_ infrared windows and left to air dry under a hood at room temperature. Likewise, standard samples were dissolved in Milli-Q water and then casted onto CaF_2_ infrared windows. Infrared microspectroscopic measurements were recorded at two beamlines: SMIS at the SOLEIL synchrotron radiation facility, France, and D7, MAX-IV laboratory, Sweden. At SOLEIL, the infrared photon source was coupled to a Thermo Fisher Nicolet Nexus 5700 FTIR spectrometer equipped with a Continuum XL microscope. A single point MCT-A detector and a 15 × 15 μm aperture were used for the measurements. At MAX-IV laboratory, the set up combined a Hyperion 3000 microscope with a Bruker IFS66/v FTIR spectrometer. The image spectra were recorded in off-line mode using a MCT focal plane array detector consisting of 128 × 128 individual detector elements. Both microscopes operated in transmission mode at 4 cm^−1^ resolution.

## Additional Information

**How to cite this article**: Lindgren, J. *et al.* Molecular composition and ultrastructure of Jurassic paravian feathers. *Sci. Rep.* **5**, 13520; doi: 10.1038/srep13520 (2015).

## Supplementary Material

Supplementary Information
