# Supplementary material for: Molecular composition and ultrastructure of Jurassic paravian feathers
Source: Sci Rep. 2015 Aug 27;5:13520. doi: 10.1038/srep13520 (PMC4550916; doi:10.1038/srep13520)
Supplement: Supplementary Information [file srep13520-s1.doc]

**SUPPLEMENTARY INFORMATION FOR**

**Molecular composition and ultrastructure** **of Jurassic paravian feathers**

Johan Lindgren^1*^, Peter Sjövall^2^, Ryan M. Carney^3^, Aude Cincotta^4,5^, Per Uvdal^6,7^, Steven W. Hutcheson^8^, Ola Gustafsson^9^, Ulysse Lefèvre^4,10^, François Escuillié^11^, Jimmy Heimdal^6^, Anders Engdahl^6^, Johan A. Gren^1^, Benjamin P. Kear^12,13^, Kazumasa Wakamatsu^14^, Johan Yans^5^ & Pascal Godefroit^4^

*^1^Department of Geology, Lund University, 223 62 Lund, Sweden. ^2^SP Technical Research Institute of Sweden, Chemistry, Materials and Surfaces, 501 15 Borås, Sweden. ^3^Department of Ecology and Evolutionary Biology, Brown University, Providence, Rhode Island 02906, USA. ^4^Operational Direction ‘Earth and History of Life’, Royal Belgian Institute of Natural Sciences, 1000 Brussels, Belgium. ^5^Department of Geology, University of Namur, 5000 Namur, Belgium. ^6^MAX-IV laboratory, Lund University, 221 00 Lund, Sweden. ^7^Chemical Physics, Department of Chemistry, Lund University, 221 00 Lund, Sweden. ^8^Department of Cell Biology and Molecular Genetics, University of Maryland, College Park, Maryland 20742, USA. ^9^Department of Biology, Lund University, 223 62 Lund, Sweden. ^10^Department of Geology, Liège University, 4000 Liège, Belgium. ^11^Eldonia, 9 Avenue des Portes Occitanes, 3800 Gannat, France. ^12^Museum of Evolution, Uppsala University, 752 36 Uppsala, Sweden. ^13^Palaeobiology Programme, Department of Earth Sciences, Uppsala University, 752 36 Uppsala, Sweden. ^14^Department of Chemistry, Fujita Health University School of Health Sciences, Toyoake, Aichi 470-1192, Japan.*

**To whom correspondence should be addressed. E-mail: johan.lindgren@geol.lu.se*

**Supplementary Tables**

**Supplementary Table S1. Mass positions, ion assignments and relative signal intensities of selected eumelanin peaks.** Observed mass positions, ion assignments and relative signal intensities of peaks related to the eumelanin molecular structure in negative ion ToF-SIMS spectra from area A1 and A2, together with synthetic and natural (*Sepia*) eumelanin. The spectra were acquired with the instrument optimised for high mass resolution. The relative signal intensities were obtained by normalising the measured intensities over the added intensities of all included peaks.


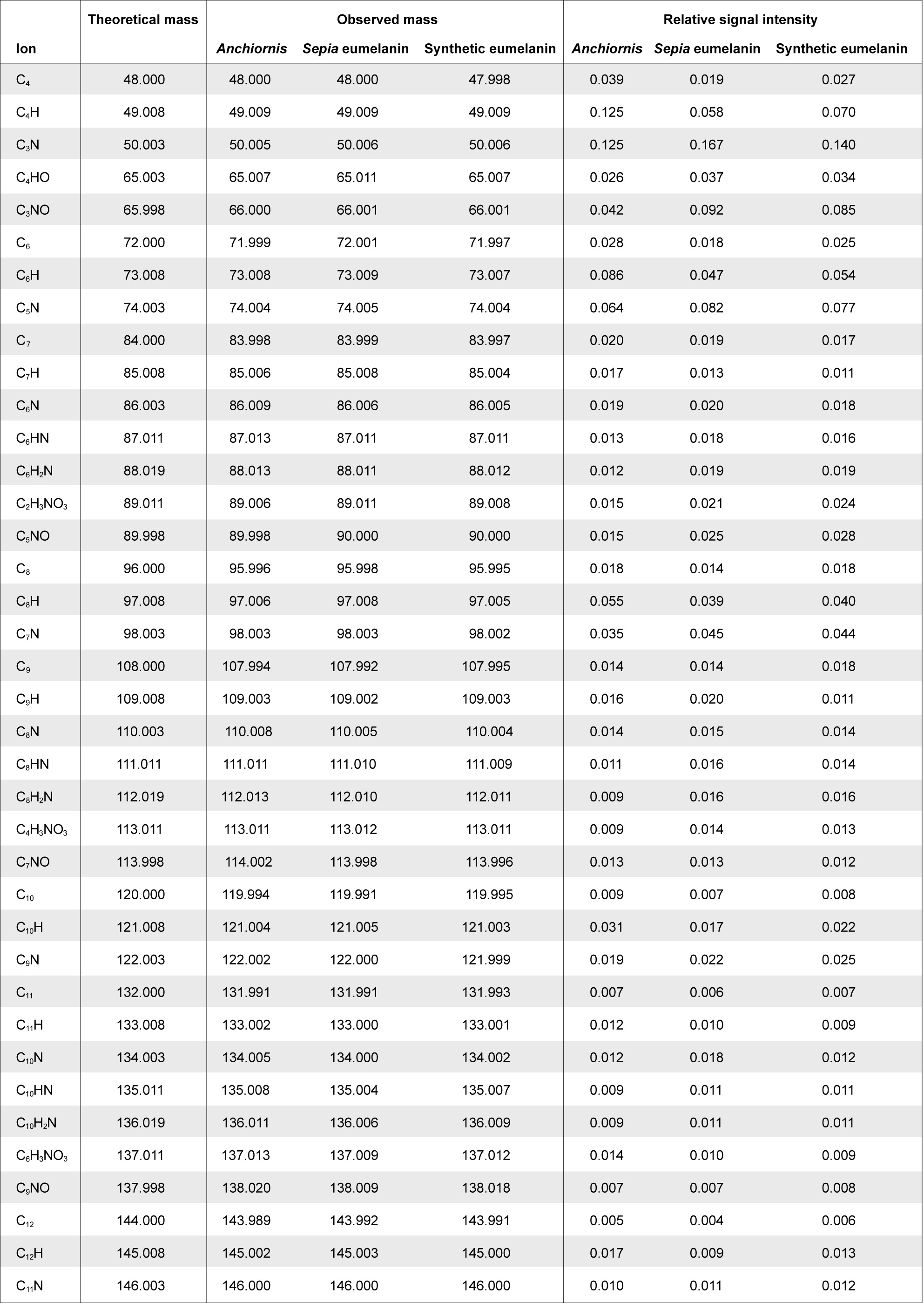


**Supplementary Table S2. Mass positions and ion assignments of non-melanic peaks in A1 and A2.** Observed mass positions and ion assignments of selected peaks that are not related to the eumelanin structure in positive and negative ion ToF-SIMS spectra from area A1 and A2.

**
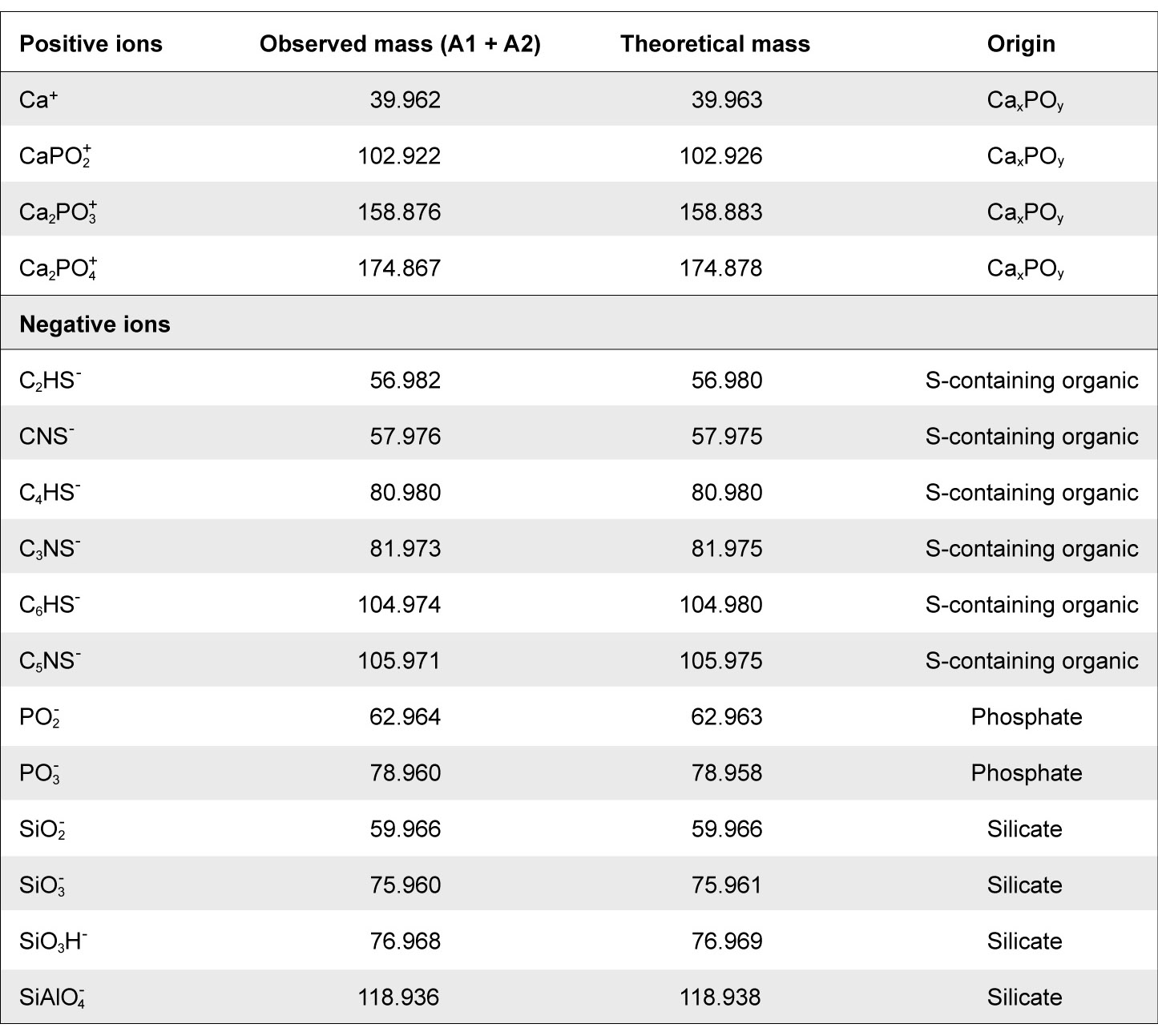
**

**Supplementary Table S3. Selected length measurements of YFGP-T5199 (mm).**

**
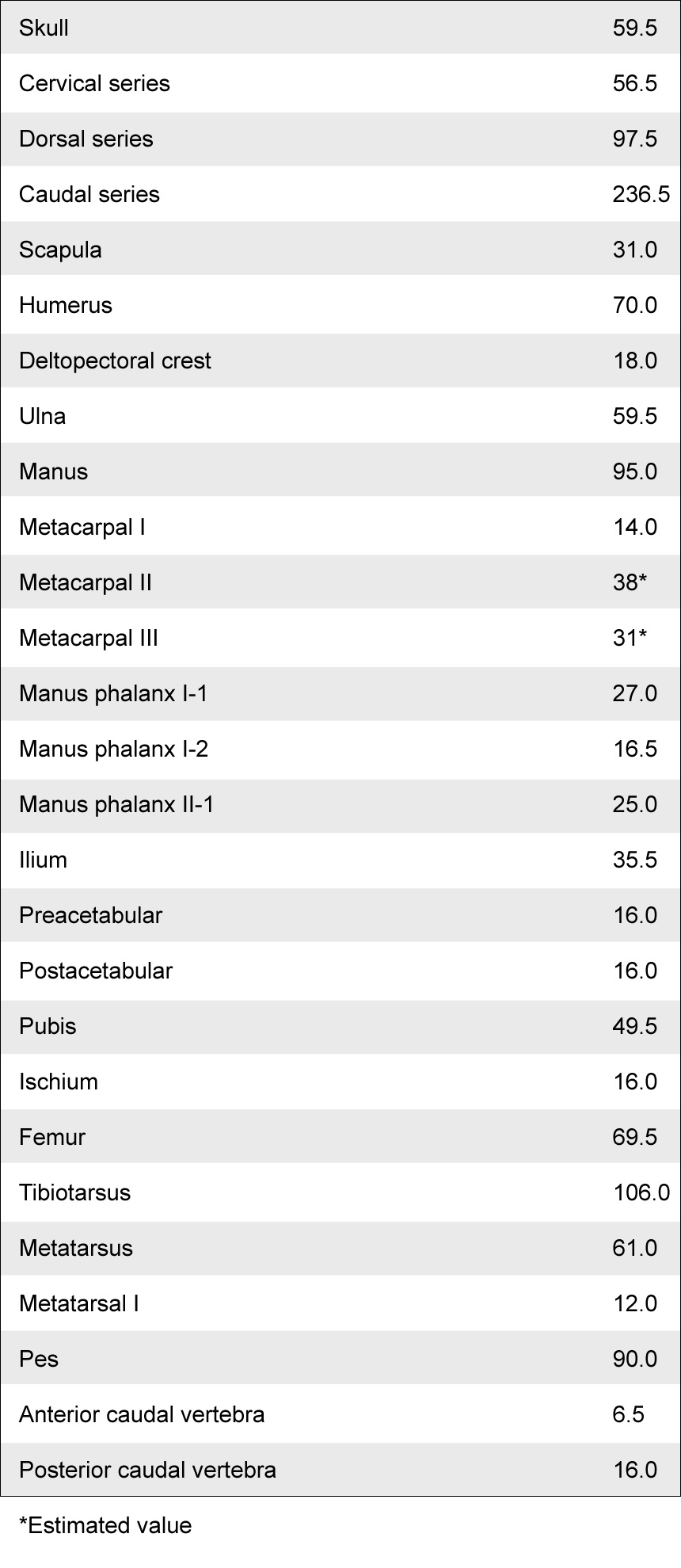
**

**Supplementary Table S4. Relative length proportions of selected skeletal elements in some Jurassic avialans.** Measurements are taken from the literature (refs 1–5,22,61).

**
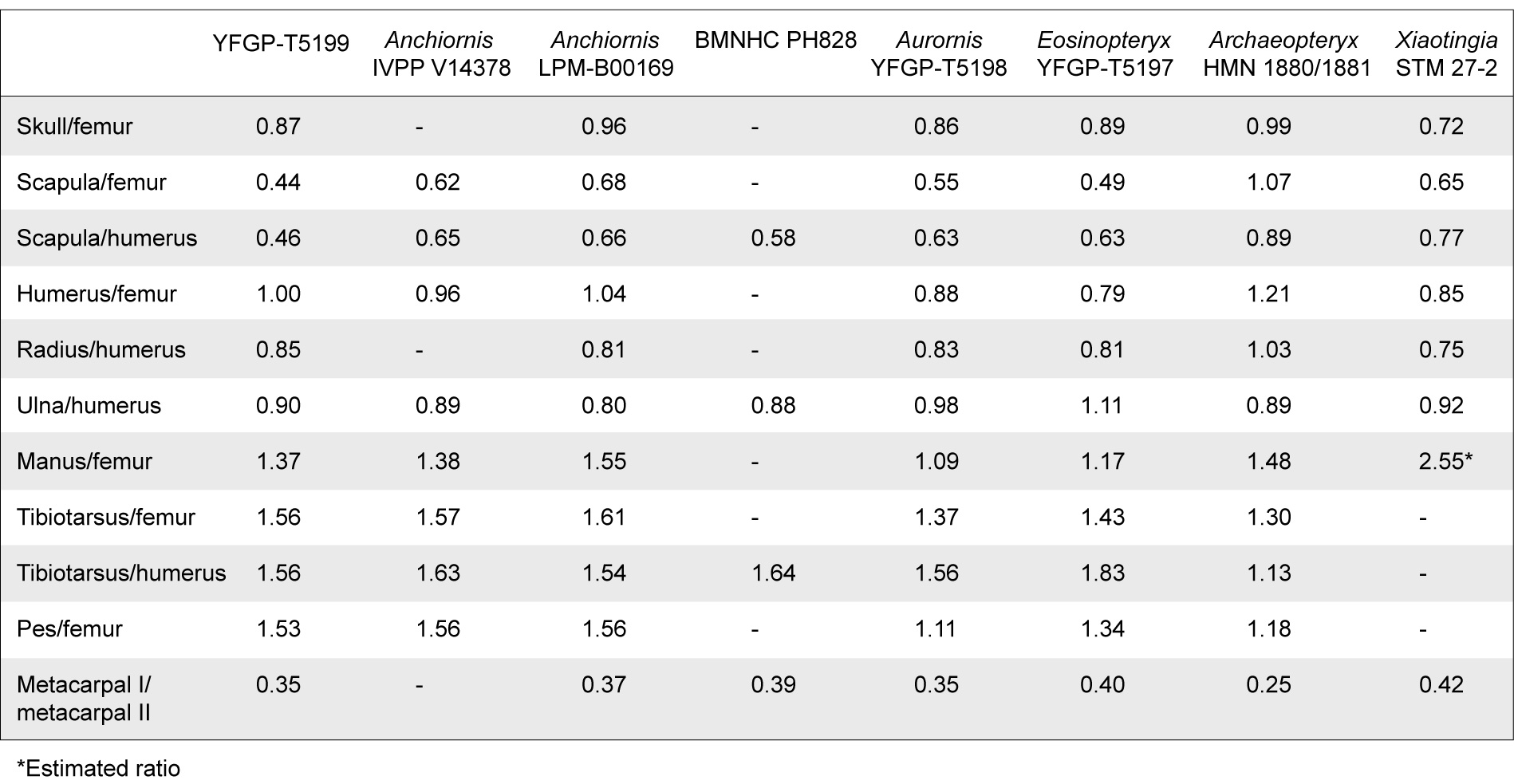
**

**Supplementary Figures**


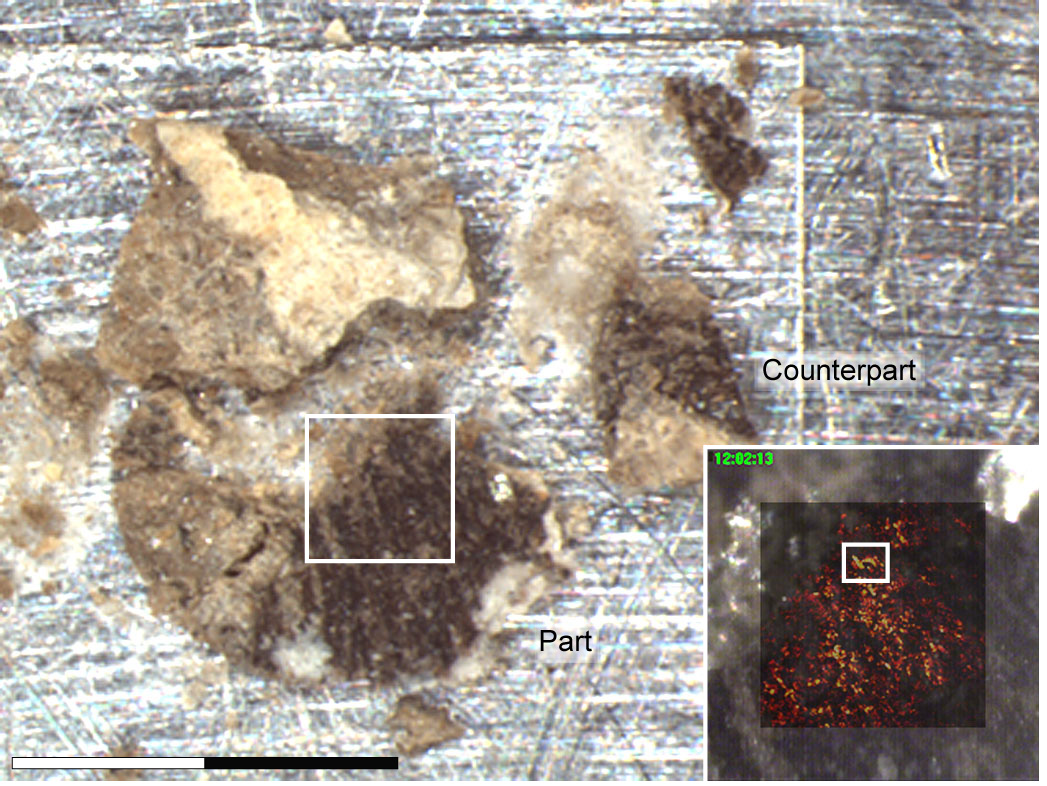


**Supplementary Figure S1. YFGP-T5199 feather sample S1.** S1 prior to molecular analysis. Note that the sample has been split into part and counterpart sub-samples. Inset shows a semi-transparent ToF-SIMS total ion image superimposed onto the demarcated area. The highlighted area in the ion image corresponds to the region analysed in Fig. 5 and Supplementary Figs S4 and S6. Scale bar: 2 mm.

**
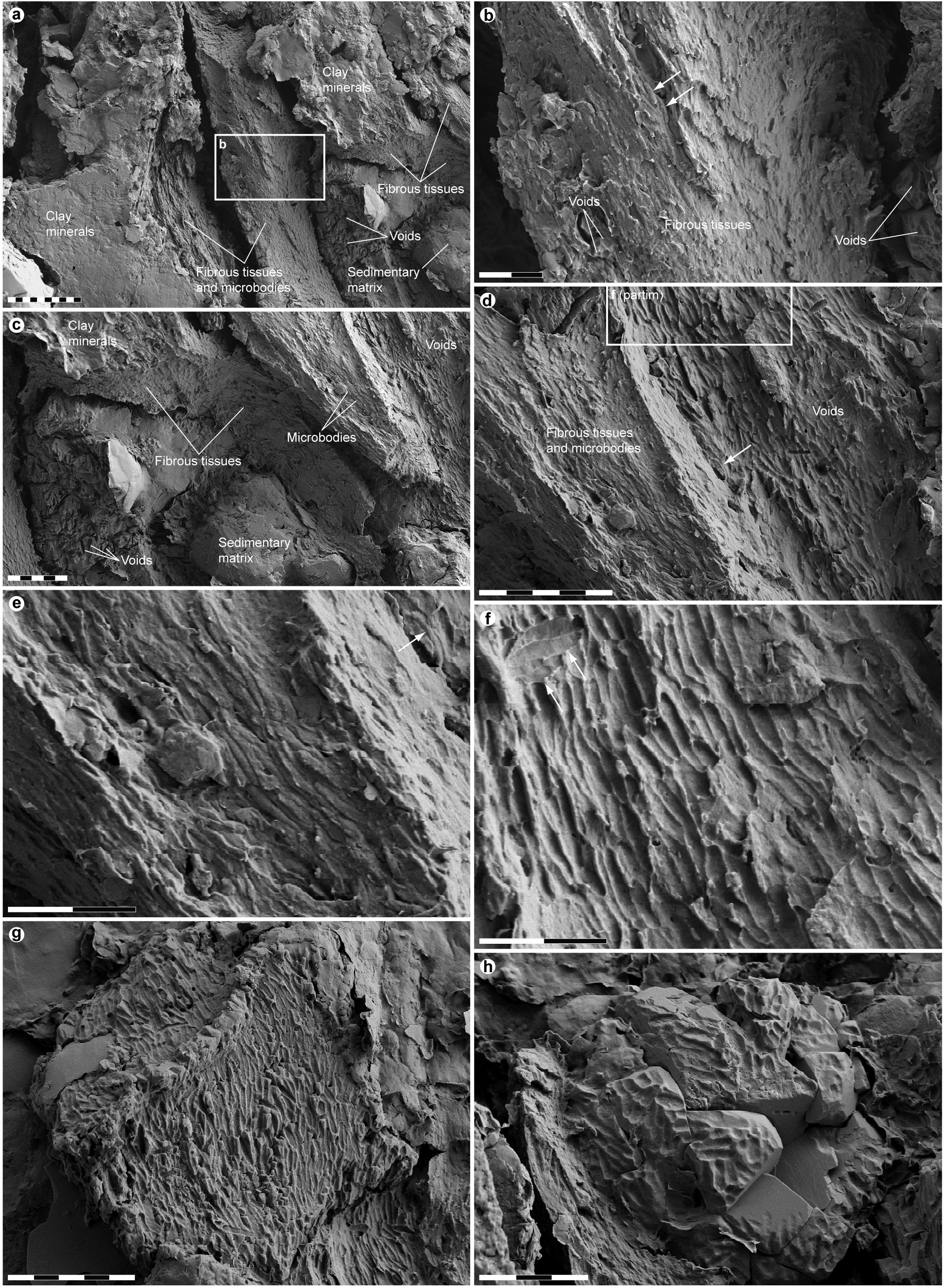
**

**Supplementary Figure S2. FEG-SEM micrographs of area A1–A7.** Close-up images of feather material and surrounding sediments in sample S1 (see also Fig. 5). (**a**) Overview of A1 and A2. Scale bar: 10 µm. (**b**) Enlargement of **a** illustrating degraded, fibril-like tissues, imprints (‘Voids’) and a few microbodies (arrows). Scale bar: 2 µm. (**c**) Overview of A3 and A5 (partim). Note transversely and longitudinally fractured fibrous tissues, intruding sedimentary matrix with scattered voids, and densely spaced microbodies. Scale bar: 5 µm. (**d**) Detail of A3 and A5 showing elongate microbodies embedded within fibril-like elements and densely spaced imprints oriented along the three-dimensional structures. Arrow indicates a microbody located in the same plane as the impressions. Scale bar: 5 µm. (**e**) Close-up image depicting eumelanosome-like microbodies within fibrous tissues. Arrow marks a partially encased microbody (see also **d**). Scale bar: 2 µm. (**f**) Enlargement of **d** demonstrating consistent orientation of imprints, albeit with some exceptions (arrows). Scale bar: 2 µm. (**g**) Overview of A6 showing regular orientation of impressions. Scale bar: 5 µm. (**h**) Overview of A7 displaying randomly arranged imprints. Scale bar: 3 µm.

**
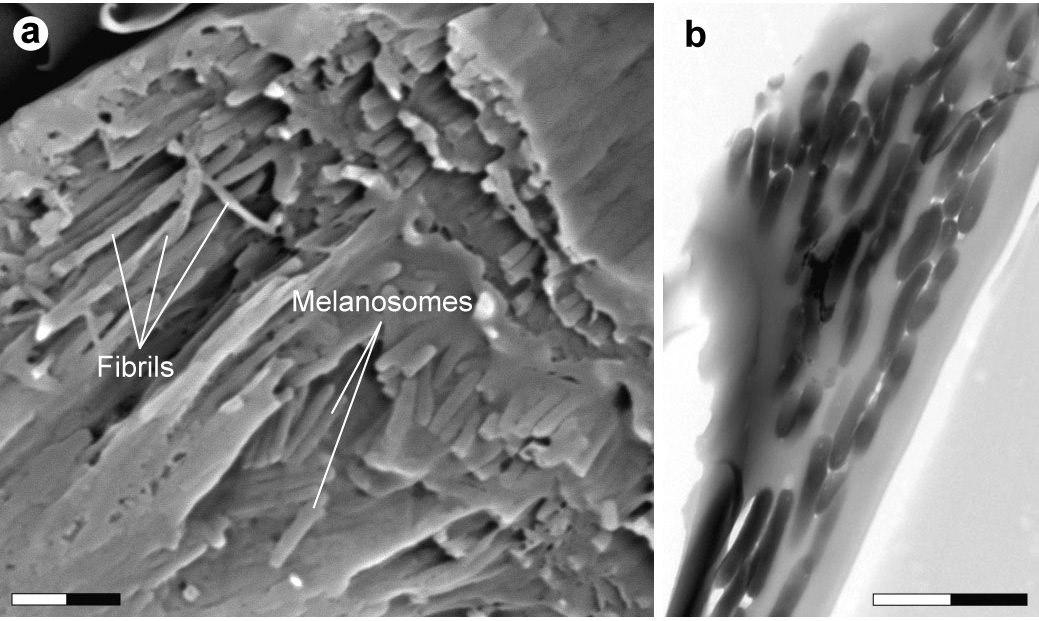
**

**Supplementary Figure S3. SEM and TEM micrographs of extant *Corvus* sp. feathers.** (**a**) Oblique transverse section of barbule. Note elongate melanosomes embedded within keratin fibrils. Scale bar: 2 µm. (**b**) TEM micrograph showing elongate melanosomes within a longitudinally sectioned barbule. Scale bar: 2 µm.

**
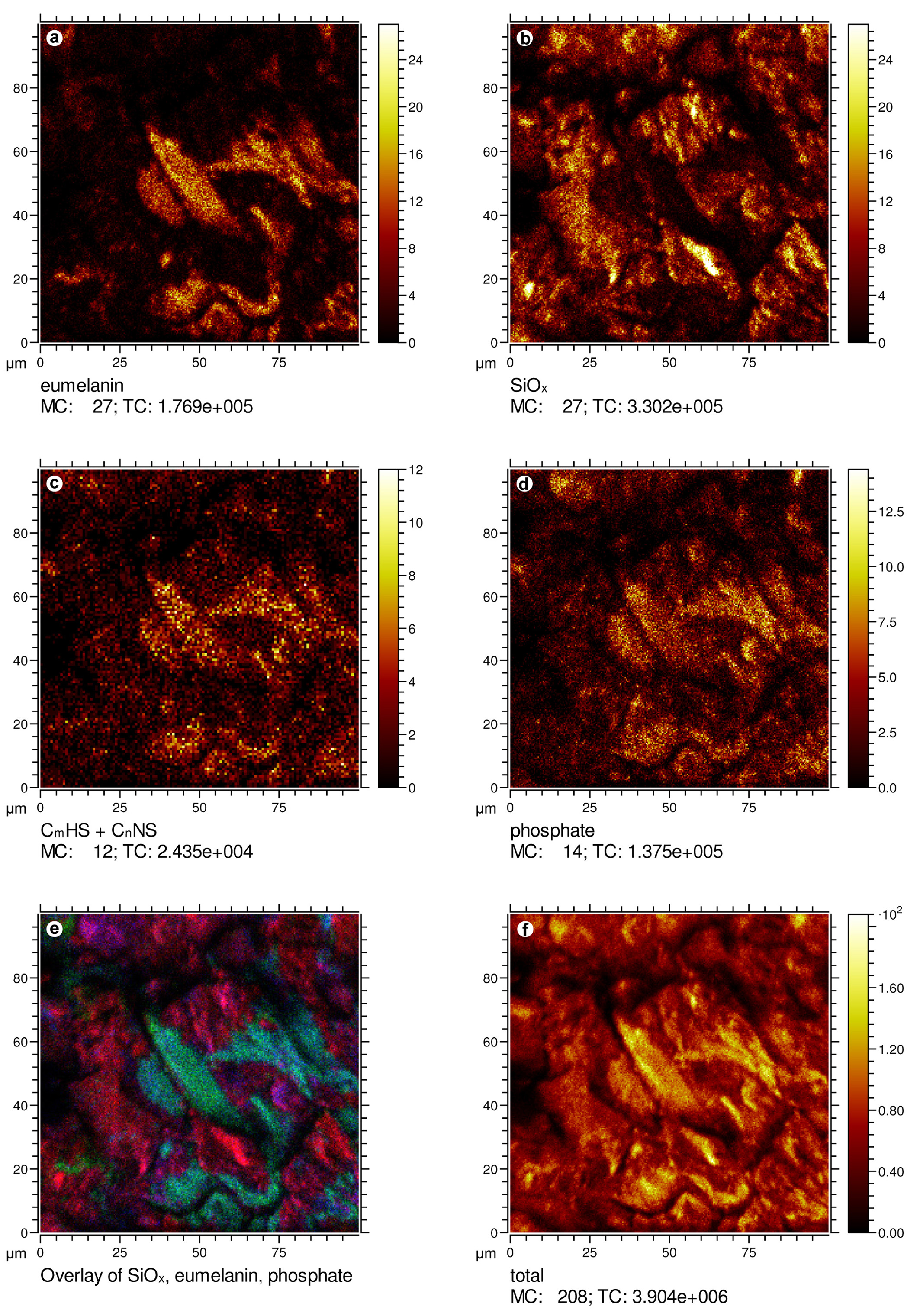
**

**Supplementary Figure S4. Negative ion ToF-SIMS images of sample S1.** Negative ion ToF-SIMS images of sample S1 showing the added signal intensity of peaks characteristic of (**a**) eumelanin (50 + 66 + 73 + 74 + 97 + 98 u), (**b**) silica (60 + 76 + 77 + 119 u), (**c**) sulfur-containing organics (57 + 58 + 81 + 82 + 105 + 106 u), and (**d**) phosphate (63 + 79 u), as well as (**e**) an overlay image of eumelanin (green), silica (red) and phosphate (blue) (same illustration as in Fig. 5c) and (**f**) the total ion image. Ion assignments are indicated under each image.

**
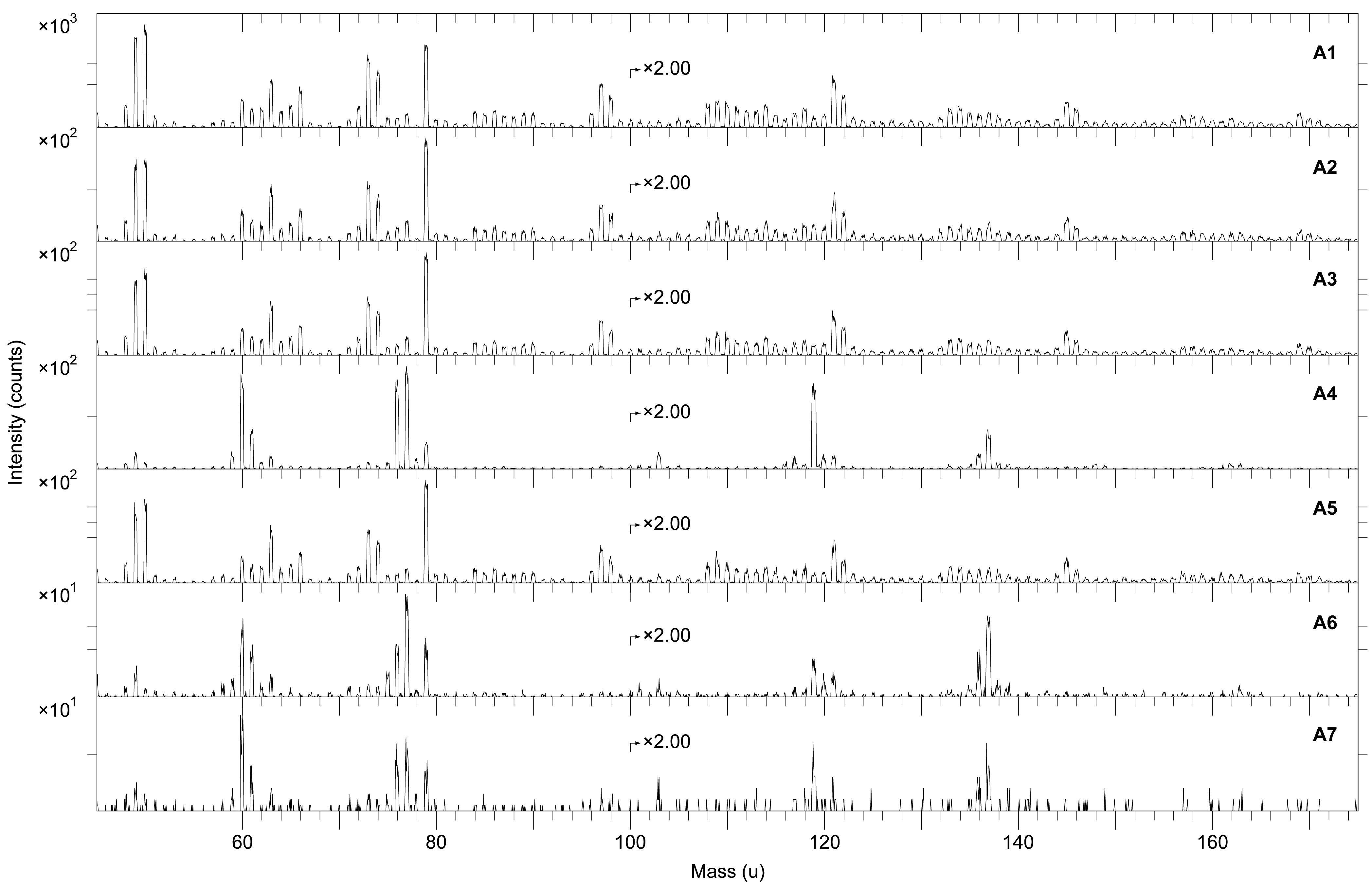
**

**Supplementary Figure S5. Negative ion ToF-SIMS spectra representing A1–A7.** Negative ion ToF-SIMS spectra obtained from area A1–A7 (Fig. 5b). Note striking resemblance between the spectra acquired from A1–A3 and A5, and the reference spectra from natural and synthetic eumelanin (Fig. 7). Peaks at 63 and 79 u correspond to phosphate ions. Also note that the spectra from A4, A6 and A7 are dominated by peaks representing silica ions. All spectra were acquired with the ToF-SIMS instrument optimised for high image resolution.

**
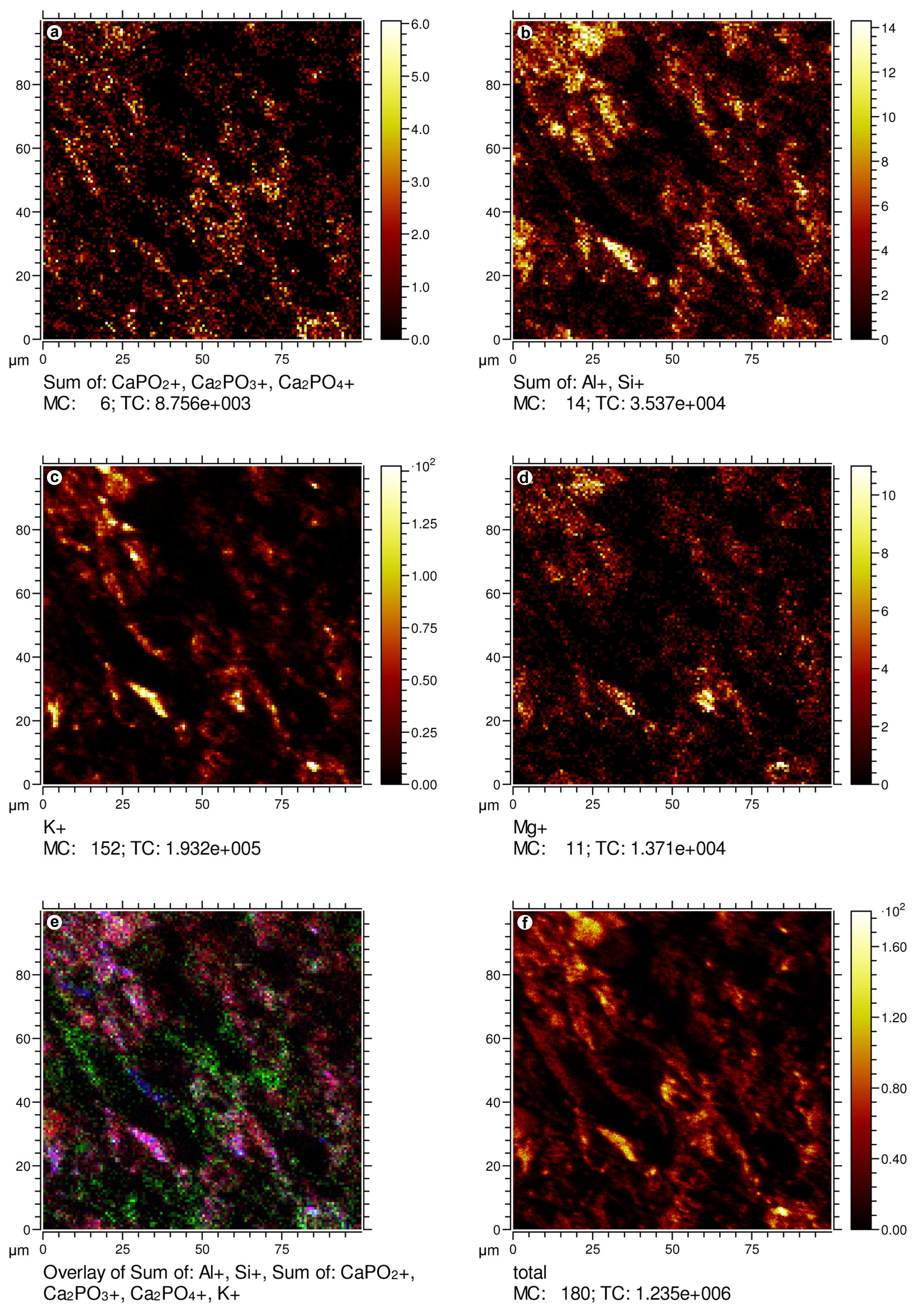
**

**Supplementary Figure S6. Positive ion ToF-SIMS images of sample S1.** Positive ion ToF-SIMS images of sample S1 showing the signal intensity of peaks characteristic of (**a**) calcium phosphate (103 + 159 + 175 u), (**b**) aluminium + silicon (27 + 28 u), (**c**) potassium (39 u), and (**d**) magnesium (24 u), as well as (**e**) an overlay image of calcium phosphate (green), aluminium + silicon (red) and potassium (blue) (same illustration as in Fig. 5d) and (**f**) the total ion image. Ion assignments are indicated under each image.

**
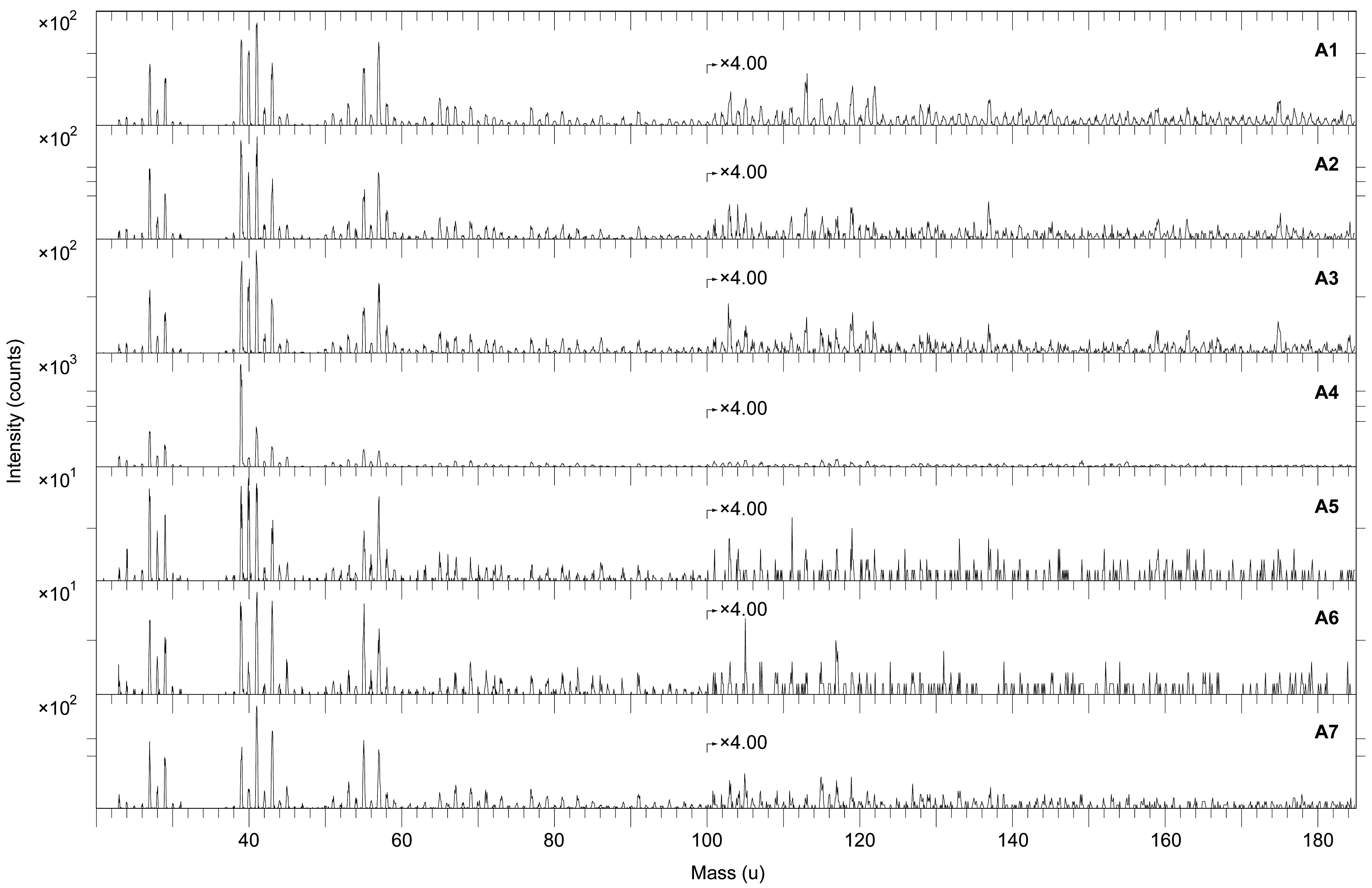
**

**Supplementary Figure S7. Positive ion ToF-SIMS spectra representing A1–A7.** Positive ion ToF-SIMS spectra obtained from area A1–A7 (Fig. 5b). Note relatively strong signal intensity from ions representing calcium (40 u) and calcium phosphate (103, 159 and 175 u) in A1–A3. All spectra were acquired with the ToF-SIMS instrument optimised for high image resolution.


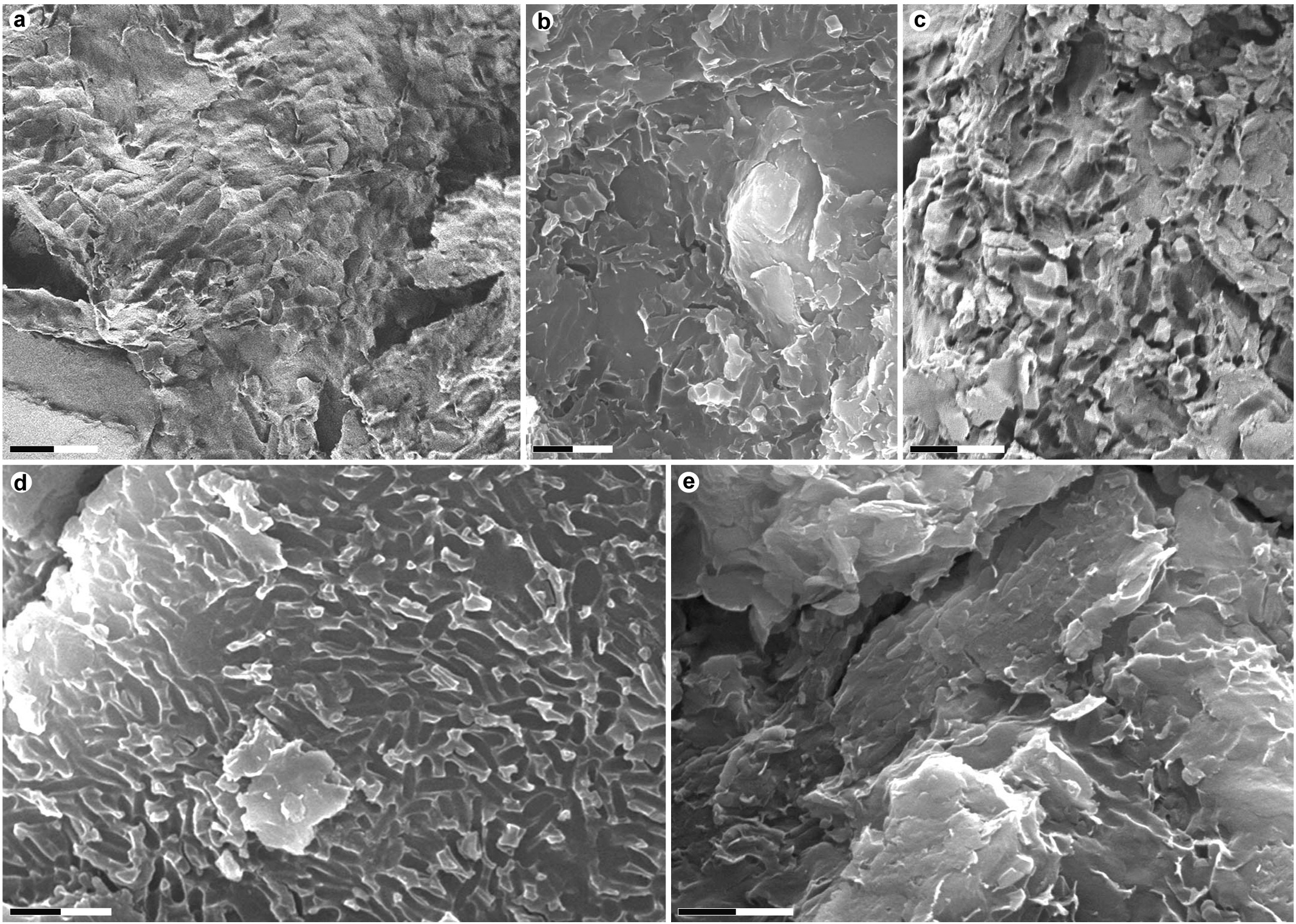


**Supplementary Figure S8. Microbodies and imprints associated with the plumage in YTGP-T5199.** FEG-SEM and SEM micrographs of (**a**) sample S5, (**b**) S11, (**c**) S12, (**d**) S13, and (**e**) S14. Scale bars: 2 µm.

**Supplementary Methods**

**Provenance and age of YTGP-T5199.** Like most paravian fossils from western Liaoning, YTGP-T5199 was acquired by Yizhou Fossil & Geology Park from a local fossil dealer, who reported that the specimen was recovered from the Yaolugou locality (Jianchang County), not far from the village of Daxishan where *Anchiornis* specimen LPM-B00169 (housed in Liaoning Paleontological Museum) was unearthed^1^. YTGP-T5199 was prepared by professional technicians at Yizhou Fossil & Geology Park, thus its authenticity and integrity (except for some minor restorations made under strict scientific control) are ensured.

The Tiaojishan Formation crops out in western Liaoning Province^62^. It is about 130–970 metres thick, and comprises a broad array of siliciclastic rocks, including basalts, andesites, rhyolites, tuffs, tuffaceous sandstones, and conglomerates^63^. Due to complex local stratigraphic conditions, accurate dating of the Tiaojishan Formation has proven difficult^64^. Absolute ages between 165 ± 1.2 and 153 ± 2.0 Ma were obtained by SHRIMP U-Th-Pb dating of samples from the nearby Lanqi Formation (a lateral equivalent to the Tiaojishan Formation)^64,65^. Zhang *et al*.^64^ concluded that the upper boundary of the Lanqi Formation ranges between 156 and 153 Ma, whereas Chang *et al*.^63^ obtained Ar-Ar ages of 160.7 ± 0.4 and 158.7 ± 0.6 Ma for the lower part of the formation. These estimates indicate that the Tiaojishan Formation spans the Callovian-Kimmeridgian interval of the Middle-Late Jurassic^66^.

**Specimen details and taxonomic assignment.** Because the holotype (IVPP V14378, housed in the Institute of Vertebrate Paleontology and Paleoanthropology) of *Anchiornis* *huxleyi* lacks a head^22^, cranial comparisons were made primarily with the virtually intact referred specimen LPM-B00169^1^.

YFGP-T5199 is about 47 cm long when measured from the tip of the snout to the distal end of the tail, and is thus comparable in size with LPM-B00169 (see Supplementary Table S3 for selected length measurements). The following features indicate that YFGP-T5199 represents a skeletally mature individual^3,67–71^: (1) the texture of the bone at the proximal and distal ends of the coracoid, humerus, ulna, pubis, and femur is regular and continuous; (2) the neural arches of the cervical and caudal vertebrae are fused to their corresponding centra; and (3) the frontals are fused along their sagittal line.

The head of YFGP-T5199 is almost complete. As in *Aurornis* and *Eosinopteryx*, it is slightly shorter than the femur^3,4^. In lateral view, the skull is sub-triangular due to its shallow snout and expanded postorbital region. These characteristics are also found in *Aurornis*, *Eosinopteryx* and *Xiaotingia*^2–4^. Similar to *Eosinopteryx* (but not *Aurornis*^3^)^4^, the maxillary process of the premaxilla is short. The external naris extends beyond the rostral border of the antorbital fenestra. This condition is similar to that of *Mei*, but contrasts with the shortened naris in *Aurornis*^3^. Similar to BMNHC PH828 (a third referred specimen of *A*. *huxleyi*^5^; but see also below), LPM-B00169, *Aurornis*, and *Eosinopteryx*, the nasal contributes to the posterior margin of the naris. The premaxillary fenestra in YFGP-T5199 is about the same size as in *Xiaotingia*, but distinctly smaller than in *Aurornis*^2,3^. YFGP-T5199 has a large maxillary fenestra separated from the antorbital fenestra by a narrow interfenestral bar, a condition comparable to that in LPM-B00169 and BMNHC PH828. Additionally, the antorbital fenestra is about half the length of the orbit, and the former cranial opening is as high as long. These features are evident in LPM-B00169, but contrast with those in *Xiaotingia* where the antorbital fenestra is considerably shorter than high^2^.

In YFGP-T5199, the posterior process of the lacrimal is shorter than the anterior process, a character state comparable to that of *Archaeopteryx* and troodontids^72–74^, but dissimilar to the long posterior process in *Eosinopteryx* and *Aurornis*^3,4^. The dentary is slender and bears a groove that widens posteriorly. It also contains a row of foramina, as in numerous other paravians, including *Archaeopteryx*, *Anchiornis*, *Xiaotingia*, *Eosinopteryx*, *Aurornis*, troodontids, and some basal dromaeosaurids^1–4,61,71^.

The tooth crowns are sharp (but not serrated), and thus unlike the bulbous teeth in *Xiaotingia*^2^. The premaxillary teeth are densely spaced in the symphyseal region, as in other basal birds (including IVPP V14378, LPM-B00169 and BMNHC PH828) and troodontids^3,4,69,74,75^. Similar to LPM-B00169, a longitudinal groove is present along the dorsomedial margin of the (slender) sub-orbital ramus of the jugal. This is unlike the condition in BMNHC PH828. This bone is also more robust than in *Aurornis*^3^.

The gross morphology of the vertebrae in YFGP-T5199 closely resembles those in basalmost Avialae, including the *A. huxleyi* holotype. The middle and posterior dorsal vertebrae are elongated, whereas the anteriormost caudals are significantly shortened. These character states manifest in other specimens assigned to *Anchiornis*, primitive dromaeosaurs, as well as other basal birds (e.g., *Archaeopteryx*, *Aurornis* and *Eosinopteryx*^1,3,4,22,61^). In the proximal caudal series, the transverse processes are posterolaterally oriented, longer than their corresponding centra, and slender; features observed elsewhere in the type specimen of *Anchiornis*. As in IVPP V14378 and *Archaeopteryx*, the distal caudals bear a distinct groove on their lateral surface near the junction between the neural arch and vertebral centrum^1^.

Similar to most troodontids, *Archaeopteryx*, *Aurornis*, *Eosinopteryx*, and all previously described specimens of *Anchiornis*^3–5,22,76,77^, there is no ossified sternum in YFGP-T5199. The furcula closely resembles that in IVPP V14378 and BMNHC PH828^1,5^. The interclavicular angle is smaller than in *Archaeopteryx*. The scapula is short and slender (approximately 46 % of humeral length), as in IVPP V14378 (65 %), LPM-B00169 (66 %), BMNHC PH828 (58 %), basal dromaeosaurids (e.g., *Microraptor zhaoianus*), and basal avians (e.g., *Aurornis*, where it is 63 %) (Supplementary Table S4; see also ref. 78). The coracoid is sub-rectangular, as in IVPP V14378, LPM-B00169, BMNHC PH828, *Aurornis*, *Eosinopteryx*, and *Archaeopteryx*. There are numerous small pits on its ventral surface, a diagnostic feature observed also in IVPP V14378, but not in the (dorsally exposed) coracoids of BMNHC PH828 and LPM-B00169^1,5^.

Similar to *Anchiornis*, *Aurornis*, *Eosinopteryx*, and *Xiaotingia*, the forelimb/hind limb ratio is below 1.0. The humeri in YFGP-T5199 equal the femora in length (1.0), and are thus comparable to those in the holotype of *A*. *huxleyi* (0.96) and LPM-B00169 (1.04), but unlike the shorter humeri in *Aurornis* (0.88), *Eosinopteryx* (0.79) and *Xiaotignia* (0.85). The deltopectoral crest is short (about one-fourth of humeral length) as in the *A. huxleyi* holotype, BMNHC PH828, dromaeosaurids, and troodontids^1,5^. The ulna is only slightly thicker than the radius and exhibits a gentle posterior curvature, as in IVPP V14378, BMNHC PH828 and *Aurornis*; this contrasts with the virtually straight ulna in *Xiaotingia* and *Eosinopteryx*^2–5,22^*.* The radius and ulna closely contact each other, unlike the condition in *Xiaotingia*, *Archaeopteryx*, *Mei*, dromaeosaurids, and *Sinornithoides*^3,79^. Metacarpal I is about one-third the length of metacarpal II, like *Aurornis*, *Eosinopteryx* and *Xiaotingia*^2–4^. Contrary to the condition in *Aurornis*, the manual phalanx I-1 is not more robust than the radius^3^. Also in contrast to *Eosinopteryx* (but similar to BMNHC PH828), metacarpal III is both as long and wide as metacarpal II^3,5^. As in the *A. huxleyi* holotype and *Eosinopteryx*, the long manual phalanx II-1 is comparable in robustness to the radius^4^, but it is also thicker than the other manual phalanges^22^.

Similar to the holotype of *Anchiornis*, the ilium of YFGP-T5199 is about half the length of the femur^1,22^. The dorsal margin of the ilium is slightly convex, as in LPM-B00169 and *Xiaotingia*, and contrasts with the sub-horizontal dorsal profile seen in *Aurornis*, and the strongly convex edge found in *Eosinopteryx*^2–4^. As in *Eosinopteryx*, the postacetabular process does not taper distally. This process remains sub-horizontal, similar to the condition in *Eosinopteryx*, *Aurornis* and *Xiaotingia*^2–4^. Although the supraacetabular crest and the prominent supratrochanteric process have been crushed during fossilisation, the general shape of the ilium in YFGP-T5199 appears to be virtually identical to that in LPM-B00169 and IVPP V14378. As in LPM-B00169 and *Eosinopteryx*, the ischium is strongly curved posteriorly with an obturator process located close to the midpoint of the element^1,4^; this is contrary to the condition in *Aurornis* where the obturator process is delimited by a hook-like ventral process and a long dorsodistal process of the ischium^3^. In YFGP-T5199, the dorsodistal process of the ischium is short and blunt, whereas it forms an elongate, tapering blade in IVPP V14378, LPM-B00169 and *Eosinopteryx*^1,4,22^. Pending additional material of *Anchiornis*, we tentatively consider this character state as representing either sexual dimorphism or intraspecific variation, since it by itself is insufficient to warrant the erection of a new species. Unlike dromaeosaurids and *Xiaotingia*, there is no groove running along the anterior margin of the ischium^2^.

The hind limbs in YFGP-T5199 are highly elongate with a femur + tibiotarsus + metatarsal III/trunk length ratio of 2.4, roughly corresponding to that in the *A. huxleyi* holotype (2.0). The distal half of the femur is thicker than the proximal half, a feature also seen in basal avialans, such as *Sapeornis* and *Rahonavis*, as well as in the primitive dromaeosaurid *Buitreraptor*^1,67,68^, but not in *Xiaotingia*, *Aurornis* and *Eosinopteryx*. The tibiotarsus is highly elongate (156 % of femoral length), as in the *Anchiornis* holotype (160 %) and LPM-B00169 (161 %). The bone is relatively longer than in any other non-avian theropod^80^ or Mesozoic avialan (Supplementary Table S4).

As in IVPP V14378, LPM-B00169, BMNHC PH828, *Aurornis*, and *Eosinopteryx*, the metatarsals are not fused proximally. Pedal digit I lies medial to metatarsal II, suggesting a non-reversed hallux, as in IVPP V14378 and LPM-B00169^1,22^. The sub-arctometatarsalian condition (that is, a transverse compression of metatarsal III) is present in YFGP-T5199, as in some dromaeosaurids and troodontids^22^, LPM-B00169, *Aurornis*^3^, and *Eosinopteryx*^4^. Metatarsal I is less than one-fourth the length of metatarsal III, as in LPM-B00169 (about 20 % in both specimens), whereas the bone is about 30 % the length of metatarsal III in *Aurornis*^3^. As in *Mei* and some dromaeosaurids, the distal articulation of metatarsal II is about as wide as the corresponding surface of metatarsal III^1^. Similar to *Archaeopteryx*, *Eosinopteryx*, *Aurornis*, *Xiaotingia*, and terrestrial cursorial birds, the pedal phalanges decrease gradually in length proximodistally^2–4,61^.

In conclusion, YFGP-T5199 closely resembles both the holotype (IVPP V14378) and referred specimen (LPM-B00169) of *A*. *huxleyi.* Diagnostic characters of *A. huxleyi* (see ref. 22) that also occur in YFGP-T5199 include: (1) numerous small pits on the ventral surface of the coracoid; (2) an extremely reduced ischium (less than one-fourth of femoral length); and (3) an elongate tibiotarsus (roughly 150 % of femoral length). Additionally, measurements and ratios presented in Supplementary Tables S3 and S4 show that the skeletal and anatomical proportions of YFGP-T5199 are virtually identical to those of IVPP V14378 and LPM-B00169, whereas they differ substantially from those of other paravians from the Tiaojishan Formation. Accordingly, YFGP-T5199 is here assigned to as *A*. *huxleyi*.

**Remarks on the systematic position of BMNHC PH828.** In 2010, Li *et al*.^5^ referred a partial paravian with extensive body coverage (BMNHC PH828) from the Daxishan site to *A*. *huxleyi*. At that time, only *Anchiornis* was known from the locality (other taxa, including *Xiaotingia*, *Aurornis* and *Eosinopteyx* have since been discovered in the area^2–4^). This stratigraphical proximity, coupled with compatible body-size (within 5 % that of the *A*. *huxleyi* holotype) and tibia length, was used to justify the taxonomic assignment.

Nevertheless, we undertook additional comparisons with IVPP V14378, LPM-B00169, and other feathered theropods from Jianchang County: *Aurornis*, *Eosinopteyx* and *Xiaotingia* (Supplementary Table S4). These revealed near identical character states, measurements and proportions between these paravians. Thus, a detailed re-examination of BMNHC PH828 is required to confirm its affinity, an undertaking that is beyond the scope of the current study.

**SEM imaging of YFGP-T5199** **plumage sample S2–S14.** The morphological dissimilarity between the microbodies and imprints in sample S1 and those previously reported from the ‘forecrown’ of another presumed specimen (BMNHC PH828) of *A*. *huxleyi*^5^ (but see also discussion above) prompted an investigation of other parts of the plumage of YFGP-T5199 (Fig. 1b). This analysis revealed that the feather traces occur in various states of preservation, ranging from pristine (as in S1) to diffuse, corroded outlines within the clay matrix. Microbodies and/or imprints were found primarily with the tail feathers, and were all oval to elongate in shape (Supplementary Fig. S8).

**Growth and preparation of eumelanin from** ***Saccharophagus degradans*.** Eumelanin was extracted from *Saccharophagus degradans* strain 2-40^81^ using the method of Banerjee *et al*.^82^. Bacteria were grown to late stationary phase in 50 ml of half strength 2216 medium. Black cells were harvested, and the pellet re-suspended in 20 ml 5 % trichloroacetic acid (TCA) for 10 min. The black precipitate was then collected via centrifugation at 8000 × g. After repetition, the pellet was washed twice in 10 ml ethanol-ether 1:1 (v:v), and once with 10 ml ether. After air-drying, the eumelanin was extracted using 50 mM NaCO_3_ at 95 ºC, and purified via gel filtration using a PD10 desalting column packed with Sephadex G25.

**Growth and preparation of pyomelanin from *Vibrio cholerae*.** Bacteria belonging to a colony of *Vibrio cholerae* strain SNW28^83^ were grown aerobically at 37 ^o^C for five days in Lysogeny broth^84^ supplemented with 25 mg of kanamycin per litre solution. Individual cells were removed from the liquid culture by centrifugation at 7500 × g for 15 min at 4 ^o^C. The supernatant was cleared by centrifugation at 50 000 × g for 30 min, resulting in a brown-coloured solution that was stored in the dark at 4 ^o^C. The pigment was purified in milli-Q water using an Amicon® ultra centrifugal filter (3kDa MWCO, Merck Millipore) at 14 000 × g for 4 × 10 min.

**Preparation of synthetic pyomelanin.** Pyomelanin was chemically derived via auto-oxidation of homogentisic acid (HGA) in accordance with the methods of ref. 85.

**Remarks on the IR data.** For our IR absorbance measurements, we used two different beamlines: SMIS at SOLEIL, France, and D7, MAX-IV laboratory, Sweden. By utilising a small area (50 × 50 µm^2^) MCT-A single element detector and the superior photon flux at SOLEIL, we were able to identify a few feather regions showing broad-band absorbance consistent with eumelanin (Fig 8a). Moreover, the set up enabled measurements of spatially small regions (15 × 15 µm^2^), thereby reducing contributions from the sediment. Two distinct peaks at 2850 and 2920 cm^-1^ (Fig. 8a) – derived from C-H stretch vibrational modes – were considered representative of melanin and used to produce the IR image in Fig. 8c. These bands were not affected by the sediment or a none-linear background (which was more pronounced in our focal plane array detector measurements).

The utility of the C-H stretches to represent residual eumelanin in fossil material is in accordance with published accounts by Glass *et al*.^42,59^ (in contrast to modern *Sepia* melanin, absorbance spectra from fossil ink sac samples exhibit well-defined and intense C-H stretches).

**Supplementary References**

61. Wellnhofer, P. Archaeopteryx, the Icon of Evolution (Dr F. Pfeil-Verlag, 2009).

62. Duan, Y. *et al*. Preliminary report on Middle Jurassic strata and fossils from Linglongta

area of Jianchang, Liaoning. *Global Geol.* **28,** 143–147 (2009).

63. Chang, S. *et al.* High-precision ^40^Ar/^39^Ar age constraints on the basal Lanqi Formation

and its implications for the origin of angiosperm plants. *Earth Planet Sci Lett.* **279,**

212–221 (2009).

64. Zhang, H., Wang, M. & Liu, X. Constraints on the upper boundary age of the Tiaojishan

Formation volcanic rocks in West Liaoning-North Hebei by LA-ICP-MS dating. *Chin*

*Sci Bull.* **53,** 3574–3584 (2008).

65. Yang, W. & Li, S. Geochronology and geochemistry of the Mesozoic volcanic rocks in Western Liaoning: implications for lithospheric thinning in the North China Craton. *Lithos*. **102,** 88–117 (2008).

66. Gradstein, F. *et al*. *A Geologic Time Scale* (Cambridge Univ. Press, 2004).

67. Forster, C. A., Sampson, S. D., Chiappe, L. M., Krause, D. W. The theropod ancestry of birds: new evidence from the Late Cretaceous of Madagascar. *Science*. **279,** 1915–1919 (1999).

68. Zhou, Z. & Zhang, Z. Anatomy of the primitive bird *Sapeornis chaoyangensis* from the Early Cretaceous of Liaoning, China. *Can J Earth Sci*. **40,** 731–747 (2003).

69. Xu, X. & Norell, M. A. A new troodontid from China with avian-like sleeping posture.

*Nature*. **431,** 838–841 (2004).

70. Turner, A. H., Makovicky, P. J. & Norell, M. A. A review of dromaeosaurid systematics

and paravian phylogeny. *Bull* *Am* *Mus* *Nat Hist*. **371,** 1–206 (2012).

71. Gao, C., Morschhauser, E. M., Varrichio, D. J., Liu, J. & Zhao, B. A second soundly

sleeping dragon: new anatomical details of the Chinese troodontid *Mei long* with

implications for phylogeny and taphonomy. *PLoS ONE*. **7,** e45203 (2012).

72. Hwang, S. H., Norell, M. A., Ji, Q. & Gao, K. New specimens of *Microraptor zhaoianus*

(Theropoda: Dromaeosauridae) from northeastern China. *Am Mus Nov*. **3381,** 1–44

(2002).

73. Xu, X., Norell, M. A., Wang, X. L., Makovicky, P. J. & Wu, X. C. A basal troodontid

from the Early Cretaceous of China. *Nature*. **415,** 780–784 (2002).

74. Makovicky, P. J. & Norell, M. A. in *The Dinosauria*, 2^nd^ edn. (eds Wheishampel, D. B.,

Dodson, P. & Osmolska, H.) 184–195 (Univ. California Press, 2004).

75. Currie, P. J. Bird-like characteristics of the jaws and teeth of troodontid theropods

(Dinosauria: Saurischia). *J Vert Paleontol.* **7,** 72–81 (1987).

76. Elzanowski, A. in *Mesozoic Birds: Above the Heads of Dinosaurs* (eds Chiappe, L. M. &

Witmer, L. M.) 129–159 (Univ. California Press, 2002).

77. Makovicky, P. J., Norell, M. A., Clark, J. M. & Rowe, T. Osteology and relationships of

*Byronosaurus jaffei* (Theropoda: Troodontidae). *Am Mus Nov.* **3402,** 1–23 (2003).

78. Makovicky, P. J., Apesteguia S. & Agnolin, F. L. The earliest dromaeosaurid theropod

from South America. *Nature*. **437,** 1007–1011 (2005).

79. Currie, P. J. & Dong, Z. M. New information on Cretaceous troodontids (Dinosauria,

Theropoda) from People’s Republic of China. *Can* *J* *Earth Sci*. **38,** 1753–1766 (2001).

80. Chrisiansen, P. & Bonde, N. C. Limb proportions and avian terrestrial locomotion. *J*

*Ornithol.* **143,** 356–371 (2002).

81. González, J. M. & Weiner, R. M. Phylogenetic characterization of marine bacterium strain

2-40, a degrader of complex polysaccharides. *Int* *J* *Syst* *Evol* *Microbiol*. **8,** 831–834

(2000).

82. Banerjee, A., Supakar, S. & Banerjee, R. Melanin from the nitrogen-fixing bacterium

*Azotobacter chroococcum*: a spectroscopic characterization. *PLoS ONE*. **9,** e84574

(2014).

83. Valeru, S. P. *et al*. Role of melanin pigment in expression of *Vibrio cholerae* virulence

factors. *Infect* *Immun*. **77,** 935–942 (2009).

# 84. Sambrook, J. & Russell, D. W. *Molecular Cloning: a Laboratory Manual* (Cold Spring

# Harbor Laboratory Press, 2001).

85.Turick, C. E., Tisa, L. S. & Caccavo, F. Jr. Melanin production and use as a soluble electron shuttle for Fe(III) oxide reduction and as a terminal electron acceptor by *Shewanella algae* BrY. *Appl* *Environ* *Microbiol*. **68,** 2436–2444 (2002).
